# Dietary Abrasiveness Is Associated with Variability of Microwear and Dental Surface Texture in Rabbits

**DOI:** 10.1371/journal.pone.0056167

**Published:** 2013-02-06

**Authors:** Ellen Schulz, Vanessa Piotrowski, Marcus Clauss, Marcus Mau, Gildas Merceron, Thomas M. Kaiser

**Affiliations:** 1 Biocenter Grindel and Zoological Museum, University of Hamburg, Hamburg, Germany; 2 Clinic for Zoo Animals, Exotic Pets and Wildlife, University of Zurich, Zurich, Switzerland; 3 Dental Institute, King's College London, Guy's Hospital, London, United Kingdom; 4 Institut de Paléoprimatologie, Paléontologie Humaine, Evolution et Paléoenvironnements, Unité Mixte de Recherche Centre National de la Recherche Scientifique 7262, Université de Poitiers, Poitiers, France; Monash University, Australia

## Abstract

Dental microwear and 3D surface texture analyses are useful in reconstructing herbivore diets, with scratches usually interpreted as indicators of grass dominated diets and pits as indicators of browse. We conducted feeding experiments with four groups of rabbits (*Oryctolagus cuniculus*) each fed a different uniform, pelleted diet (lucerne, lucerne & oats, grass & oats, grass). The lowest silica content was measured in the lucerne and the highest in the grass diet. After 25 weeks of exposure to the diets, dental castings were made of the rabbit's lower molars. Occlusal surfaces were then investigated using dental microwear and 3D areal surface texture analysis. In terms of traditional microwear, we found our hypothesis supported, as the grass group showed a high proportion of (long) “scratches” and the lucerne group a high proportion of “pits”. Regardless of the uniform diets, variability of microwear and surface textures was higher when silica content was low. A high variability in microwear and texture analysis thus need not represent dietary diversity, but can also be related to a uniform, low-abrasion diet. The uniformity or variability of microwear/texture analysis results thus might represent varying degrees of abrasion and attrition rather than a variety of diet items per se.

## Introduction

Plant phytoliths (opaline silica and plant silica) are considered defenses against herbivory because they decrease the digestibility of plant components and abrade mammalian teeth due to the greater hardness of phytoliths compared to enamel [1], [2], although the question whether they are actually harder than enamel has not been resolved conclusively to date [3], [4]. Herbivory by voles, for example, induced silica incorporation in grasses [5] and reduced growth rates and digestion efficiency of the voles [6]. Field studies indicate that ingested soil abrades teeth in ungulates [7], [8]. Therefore, the importance of external abrasive particles such as soil, dust, sand, and grit [9] as causative factors for tooth abrasion in ungulates has received significant attention. Phytoliths might have a greater role in causing variation in tooth wear [8]. But until now, apart from a feeding experiment of Baker et al. [1] testing for the wear of sheeps' incisors due to soil ingestion, controlled feeding experiments testing the role of phytoliths on tooth wear, and in particular on the microwear patterns of molar surfaces, are still missing.

In most mammalian groups it is assumed that the consumption of grass, which is rich in phytoliths, causes a two-dimensional microwear pattern of mainly scratches, while ingestion of browse, and hard items like seeds, produces pits [10]–[12]. These patterns were also observed in rodents, with typical grazers presenting an abundance of scratches, while insectivores and frugivores display more pits [13]–[16].

Since the traditional dental microwear analysis is limited to two dimensions, surface texture analysis, a more quantitative three-dimensional (3D) method, has become established for dental dietary reconstruction [17]–[20]. Most such studies are based on museum specimens and information on the natural diet of a species is usually taken from the literature. Comprehensiveness, accommodation of effects of seasonality and habitat conditions covered, as well as methods applied result in inconsistency of data provided. Only a few reports are based on long term observation and provide data with strong statistical evidence. For most species, it is nevertheless possible to assign a general dietary strategy that allows understanding occlusal surface features that are related to this dietary strategy. However, we do not know how much variability in diet is reflected by occlusal surface textures, nor the exact mechanism of texture formation.

This gap of knowledge in relating textures to actual diets led us to conduct feeding experiments with controlled diets of known silica content, and relate them to occlusal parameters. The study thus aims at quantifying surface textures and to relate them to the proportions of abrasives (silica) in the diet. This should enhance our understanding of the equilibrium between abrasion and attrition (e.g. [21]) in mammal post canine teeth. Abrasion is due to food-tooth or particle-tooth contact while attrition is due to (most closely) tooth-tooth contact [22], [23]. Teaford and Walker [24] showed that tooth-tooth wear produces featureless surface in stillborn guinea pigs. We would not expect to see the extreme featureless surface in the rabbits. Since Rensberger [25] recognised that food is involved in most or perhaps all natural wear and Fortelius [26] concluded that most dental wear resulted from abrasion, we used the domestic rabbits as a model species to measure high abrasion- as well as low abrasion-induced textures.

If theoretical attritional tooth-tooth contacts happen, we assume that surface structures like spiky summits would be moved against each other and it is likely that plateau-like surface structures will result, which can be measured. The fact that the ever-growing molars of the rabbits have high wear rates as compared to ungulates and primates [27] reduces the risk of cumulative wear signatures. The ISO/FDIS parameters (ISO/FDIS25178, [28]) applied to texture models allow quantification of aspects of the basic geometry of surface textures and comprehensively indicate their biomechanical properties in ungulates [19] and primates [17]. We expect our results to contribute to the better understanding of surface textures of lagomorphs and occlusal surface functionality of other species with hypselodont check teeth. Furthermore, we expect new insights into biomechanical constraints of the general process of tooth wear in mammals.

As compared to high silica diets, we assume that low-silica diets have a lower probability to induce surface lesions. Also, from microwear experience we expect point lesions (“pits”) to prevail over linear lesions (“scratches”). Overall, random effects should increase in surface textures. We thus test the following hypotheses: A higher proportion of silica particles in the grass meal feed causes a scratch-dominated pattern of comparatively low variability; a lower proportion of silica particles in the lucerne feed causes a pit-dominated and more variable surface texture.

## Materials and Methods

Thirty-two New Zealand white rabbits were kept in 4 groups receiving exclusively 4 different pelleted compound feeds consisting of grass meal (G, n = 7), grass meal with crushed oats (GO, n = 6), lucerne with crushed oats (LO, n = 7) and lucerne (L = 6) [17], [19], [29]. Water was available ad libitum. Pellets are different from a natural diet in terms of physical properties and lower water content than fresh plant material. This might have an influence on the wear characteristics, but to date, no information on the influence of feeding the same diet fresh (as forage in the wild), dried (as hay) or pelleted is available. Nevertheless, pelleted food offers the advantage of large batches of a uniform feed of consistent composition, where the influence of selective feeding on the part of the animal is excluded. Oats were used as an additive to create diets of intermediate silica content.

Coprophagy was not prevented, in order to allow wear to develop as would be representative for free-ranging animals. Gidenne and Lebas [30] observed that rabbits just swallow the soft pellets (cecotrophs) without chewing. This observation is supported by the frequent observation that cecotrophs are found in the stomach of slaughtered rabbits intact, i.e. without indication of mechanical disruption (an observation also made at the end of this experiment; M. Clauss, pers. obs., see Fig. S1 in File S1). We therefore do not expect coprophagy in rabbits to influence dental surface textures. New Zealand white rabbits (Leporidae, Lagomorpha) are a breed of the common rabbit *Oryctolagus cuniculus*. They are essentially intermediate feeders in the wild [31]. After 25 weeks animals were sacrificed, skulls were macerated, and moulds were made from the lingual part of the mesial enamel ridge of the lower first molars.

Diets were analyzed for silica concentration using a dry ash method [32]. Dental microwear analysis [20] was conducted following the protocol by Merceron et al. [33] as adapted to small mammal teeth by Gomes Rodrigues et al. [13]. A stereomicroscope (Leica MZ 16) with a spot CCD camera (Leica DC 300) at ×100 magnification was used. A 300×300 µm square was outlined on the centre of the dental facet. Microwear features *Np* (number of pits), *Np10* (number of pits >10 µm), *Np5* (number of pits >5 µm), *Ns* (number of scratches), *Nws5* (number of scratches wider than 5 µm), *Nws10* (number of scratches wider than 10 µm), *Ls* (length of scratches) were counted by Vanessa Piotrowski (one observer only) using Optimas 6.2 software (Media Cybernetics, Rockville, U. S.).

As a second method of dental wear analysis at micro scale the 3D areal surface texture analysis was conducted using the confocal disc-scanning system µsurf custom (Nanofocus AG, Germany) according to Schulz et al. [19]. Threshold of recorded points was set to 80% as compared to a threshold of 90% in Schulz et al. [19]. For both methods, the measuring area is the lingual enamel facet of the primary shearing blade of the first lower molar. Before 3D surface texture parameters can be applied to surface data, however, filtering operators should be employed after ISO/FDIS 25178 [28]. The default operator is the set of S-Filters. As a default, the areal Gaussian filter (one of the S-Filters) is applied, which excludes the smallest scale elements from the surface resulting in the primary surface. In order to suppress form alterations (e.g., the curvature of a cylinder), the F operator is applied, which results in the S-F surface [28]. Subsequently, the L-Filter removes the low frequency alterations. The final product is the S-L surface. In accordance with Schulz et al. [19], Calandra et al. [17] and Winkler et al. [34] we apply the 3D ISO/FDIS 25178 texture parameters on the S-F surface. In order to determine whether and in what way form and waviness of the tooth enamel influences our results, we additionally apply the texture parameters on the primary as well as the S-L surface.

**Figure 1:**
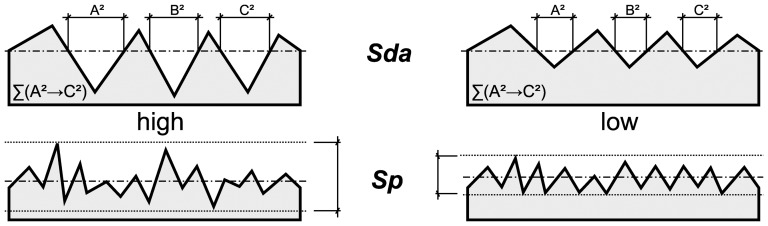
Schematic models. Schematic models of hypothetical surface textures indicating the parameter value of the ISO/FDIS 25178 parameter closed dale area (*Sda*) or maximum peak height (*Sp*) having high (left) or low values (right).

The 3D ISO/FDIS 25178 texture parameters (Table 1) employed to quantify texture were originally developed to classify 3D areal surface textures [28] to get an understanding of how the topography was influenced by industrial manufacturing processes and how the topography influences its function [35]. In ungulates, the ISO/FDIS 25178-2 parameters *Sq* (height amplitude of the surface), *Sal* (autocorrelation length), *Shv* (closed hills volume), *Spd* (density of peaks) and *Std* (texture direction) have been demonstrated to be indicative of dental functional traits [19]. Purnell et al. [36] demonstrated that the ISO/FDIS 25178-2 parameters *Sa* (mean surface roughness), *Sk* (roughness depth of the core), *Spk* (roughness depth of the peaks), *Vmp* (material volume of the peaks), *Vmc* (material volume of the core), and *Vvc* (void volume of the core) reflected diet in cichlid fishes. In primates, the ISO/FDIS 25178-2 parameters *Sq* (height amplitude of the surface), *S5v* (depth of the valleys), *Vm* (material volume), *Spd* (density of peaks), *Sha* (closed hill area), and closed dale area (*Sda*) allows inference on functional interaction of food and tooth enamel during mastication [17]. Generalised schematic models of the surface textures with high and low values of closed dale area (*Sda*) or height amplitude of the surface (*Sp*) are given in Figure 1.

**Table 1:** Description of the ISO/FDIS 25178 parameters.

Parameter	Description (condition)	Unit
*S10z*	ten-point height	µm
*S5p*	five-point peak height	µm
*S5v*	**five-point valley height**	µm
*Sa*	arithmetic mean height or mean surface roughness	µm
*Sal*	auto-correlation length (s = 0.2)	µm
***Sda***	**closed dale area**	**µm^2^**
***Sdq***	**root mean square gradient**	**no unit**
***Sdr***	**developed interfacial area ratio**	**%**
*Sdv*	**closed dale volume**	**µm^3^**
*Sha*	closed hill area	µm^2^
*Shv*	closed hill volume	µm^3^
*Sku*	kurtosis of the height distribution	no unit
*Smc*	inverse areal material ratio (p = 10%)	µm
*Smr*	areal material ratio, bearing area ratio at a given height (c = 1 µm under the highest peak)	%
***Sp***	**maximum peak height, height between the highest peak and the mean plane**	**µm**
*Spc*	arithmetic mean peak curvature	1/µm
*Spd*	density of peaks	1/µm^2^
*Sq*	standard deviation of the height distribution, or RMS surface roughness	µm
***Ssk***	**skewness of the height distribution**	**no unit**
*Std*	texture direction	°
*Str*	texture aspect ratio (s = 0.2)	no unit
*Sv*	maximum pit height, depth between the mean plane and the deepest valley	µm
***Sxp***	**peak extreme height difference in height between p% and q% (p = 50%, q = 97.5%)**	**µm**
***Sz***	**maximum height, height between the highest peak and the deepest valley**	**µm**
*Vm*	material volume at a given material ratio (p = 10%)	µm^3^/µm^2^
*Vmc*	material volume of the core at given material ratio (p = 10%, q = 80%)	µm^3^/µm^2^
*Vmp*	material volume of peaks (p = 10%)	µm^3^/µm^2^
*Vv*	void volume at a given material ratio (p = 10%)	µm^3^/µm^2^
*Vvc*	void volume of the core (p = 10%, q = 80%)	µm^3^/µm^2^
***Vvv***	**void volume of the valley at a given material ratio (p = 80%)**	**µm^3^/µm^2^**

Description and units of the applied parameters are indicated according to ISO/FDIS 25178 analysis. The most effective parameters that are found to discriminate rabbit diets are set in bold.

Statistical analyses were performed using the software R 2.12.1 [37]. The Welch-Yuen heteroscedastic omnibus test (WY [38], [39]) was coupled with a heteroscedastic pair-wise comparison test (analog to Dunnett's T3 test, PW [40]) to detect differences between trimmed means (15% trimming). Additionally the heteroscedastic rank-based test after Cliff (CM [40]) was applied. Only texture patterns found significant (*p*≤0.05) in both robust approaches were described and discussed. Discriminant analysis was applied in SYSTAT 12 (SYSTAT Software, Inc., Chicago, U.S.). The automatic stepping forward algorithm was employed using the values F_to enter_ = 1 and F_to remove_ = 0.9. Group centroids were calculated with confidence intervals of 90%.

## Results

**Figure 2:**
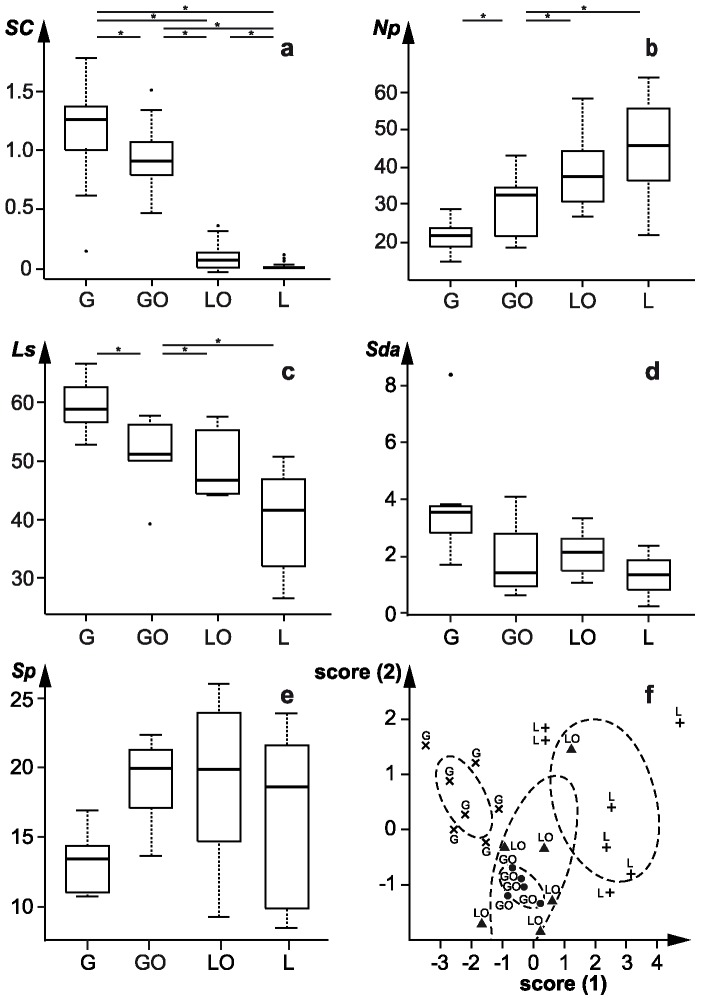
Box plots and discriminant analysis plot. Box plots indicating (a) the silica concentration (SC, %) in animal feeds, the microwear parameters (b) number of pits (*Np*), (c) length of scratches (*Ls*), the ISO/FDIS 25178-2 parameters: (d) closed dale area (*Sda* on primary surface, µm^2^), (e) maximum peak height (*Sp* on S-L surface, µm) within the feeding groups (G = grass meal, GO = grass meal with crushed oats, LO = lucerne with crushed oats, and L = lucerne), * = *p*≤0.05. Box plots showing the median (middle line), the interquartile range (IQR, box) and the minimum/maximum values 1.5×IQR (whiskers), extreme values are excluded. Discriminant analysis (f) using microwear (*Np*, *Ls*) and texture parameters (*Sda* on primary surface, *Sp* on S-L surface) indicating significant group differences (Table 2). The canonical discriminant function coefficients are *Np*_score1/2_ = 0.038/0.015, *Ls*_score 1/2_ = −0.128/−0.014, *Sda*_score1/2_ = −0.532/0.015, *Sp*_score 1/2_ = −0.076/−0.226.

Silica contents differed significantly between all groups (*p*<0.001; Fig. 2A, Table 2), ranging from 11.96 mg/g dry matter in the grass to 0.10 mg/g dry matter in the lucerne group. Group G had the lowest number of pits (*Np*) and the longest scratches (*Ls*) (Fig. 2B–C, Table 2 and Table S1, S2, S5A and S6A in File S1). The reverse was found for Group L. The variation in number of pits (Fig. 2B) and length of scratches (Fig. 2C) indicated by the interquartile range of the box plots was lower when dietary silica content was higher (L>LO>GO>G). The differences in the means of *Np* values between grass-fed (*Np*_grass-fed lagomorphs_ = 21.7) and lucerne-fed lagomorphs (*Np*_lucerne-fed lagomorphs_ = 45.9) are similar to the ones between grazing and browsing ungulates (cf. mean values of *Np*_grazing ungulates_ = 15, *Np*_browsing ungulates_ = 31, [41]). However, the differences in *Ls* values between grass-fed (*Ls*_grass-fed lagomorphs_ = 59.1 µm) and lucerne-fed lagomorphs (*Ls*_lucerne-fed lagomorphs_ = 38.9 µm) are slightly smaller than the ones reported in ungulates (*Ls*_grazing ungulates_ = 170.4 µm, *Ls*_browsing ungulates_ = 139.6 µm, [41]).

**Table 2:** WY-test statistics.

	Parameter	Ft	*p*	nu1	Nu2
silica	*SC*	386.714	<0.001	3	61.145
microwear (A)	*Np*	8.894	0.004	3	9.166
	*Ls*	6.571	0.011	3	9.547
texture (A)	*Sda*	4.359	0.037	3	8.971
	*Sdv*	5.971	0.016	3	9.128
texture (B)	*Sz*	2.972	0.095	3	8.213
texture (C)	*S5v*	5.474	0.021	3	8.868
	*Sal*	4.445	0.035	3	9.043
	*Sp*	4.192	0.043	3	8.629
	*Ssk*	4.498	0.034	3	9.169
	*Sv*	3.862	0.048	3	9.424
	*Sz*	4.282	0.042	3	8.432

Test statistics from WY-tests with 15% trimming for the silica concentration, microwear (primary surface only) and texture analyses of the primary surface (A), S-F surface (B), and S-L surface (C).

Values in bold indicate a significant difference (*p*≤0.05). *Ft* = test statistics, nu1 and nu2 = 1^st^ and 2^nd^ degree of freedom, *p* = significance level, *SC* = silica concentration [%] in animal feeds, *Np* = number of pits, *Ls* = length of scratches (µm), *Sda* = closed dale area [µm^2^], *Sdv* = closed dale volume [µm^3^], *S5v* = five point pit height [µm], *Sal* = auto correlation length [µm], *Sp* = maximum peak height [µm], *Ssk* = skewness, *Sv* = maximum pit height [µm], *Sz* = maximum height [µm].

**Figure 3:**
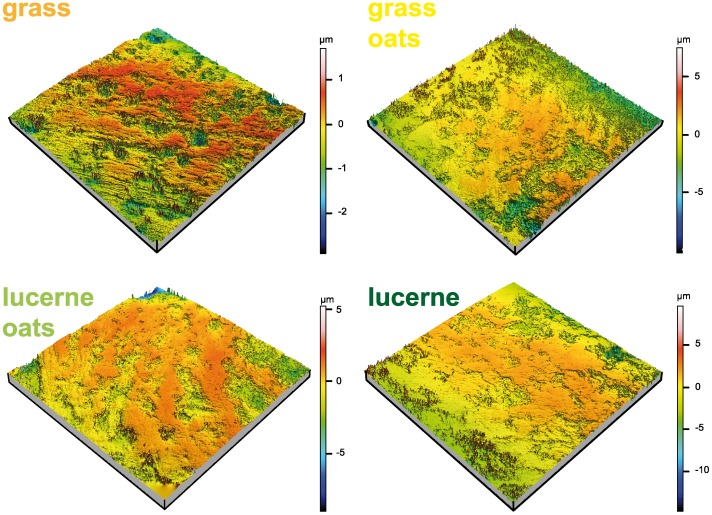
Meshed 3D models. Meshed axiomatic 3D models of tooth enamel surfaces (primary surface) of the second upper molar (160×160 µm) of animals feeding on grass (UZH-G8), grass/oats (UZH-GO4), lucerne/oats (UZH-LO6), or lucerne (UZH-L2). A deeper red indicates a top of a plateau or hill, whereas a green towards dark blue indicates deeper areas.

Group G had large areas of surface lesions (high *Sda*) on the primary surface and lower and less variable mean peak heights values on the S-L surface (low *Sp*, Fig. 2D–E, Table 2 and Tables S1, S3, S4, S5B, S6B in File S1). Group L was characterised by low *Sda* on the primary surface and higher variability in *Sp* on the S-L surface (Fig. 2D–E). Groups GO, LO and L group had similar mean *Sda*, indicating that both lucerne and the inclusion of oats lowered the probability of surface lesions (Fig. 3). Table S1 in File S1 summarises the group-wise and pair-wise comparisons, and detailed test results for all variables are given in Tables S4, S5, S6 in File S1. Silica, microwear, and 3D texture parameters for the primary, S-F, and S-L surface indicate that beside silica concentration (*SC*) and microwear parameters (*Ls*, *Np*) the 3D texture parameters *Sda* (primary surface) and *Sp* (S-L surface) are most significant in differentiating the feeding group GO from G and L. Apart from *Sda* and *Sp*, the parameters *Sdv* (primary surface), *Sdq*, *Sdr*, *Sz* (S-F surface) and *S5v*, *Sal*, *Ssk*, *Sdr*, *Sdq*, *Sv*, *Sxp*, *Sz* and *Vvv* (S-L surface) describe single group differences (Table S1 in File S1). In the ISO/FDIS 25178 the parameters are pooled in height, spatial, hybrid, function and segmentation parameters [28]. We found that on the primary surface, parameters (*Sda*, *Sdv*) derived from segmentation parameters dividing the surface into motifs (dales and hills) yield prominent differentiation in our case, while on the S-F surface, a height (*Sz*) as well as two hybrid parameters (*Sdr*, *Sdq*) related to the spatial shape of the surface yield such differentiation. Only on the S-L surface all parameter groups (height (*Sp*, *Ssk*, *Sz*, *Sv*), spatial (*Sal*), hybrid (*Sdr*, *Sdq*) and function (*S5v*, *Sxp*, *Vvv*) yield such differentiation.

The discriminant analysis using the most significant microwear (*Ls*, *Np*) and texture parameters (*Sda* on primary surface, *Sp* on S-L surface) indicates that G and L occupy distinct factor spaces, while groups GO and LO overlap (Fig. 2F). The factor space of both groups which include grass meal (G, GO) was smaller than that for lucerne, which indicates a specific, consistent texture pattern, while the groups with lucerne (LO, L) showed a larger factor space which indicates higher variability in surface texture.

## Discussion

Two-dimensional microwear [1], [12], [13], [16], [42]–[47] as well as the three-dimensional microwear texture (scale sensitive fractal analysis, [11]) method represent established approaches for dietary reconstructions in mammals [11], [20], [48]–[51]. More recently, ISO parameters became available for such dietary reconstruction [21] and are used in dietary discrimination of fishes [36] and functional analysis of complex dental surfaces in ungulates [19] and primates [17]. Our controlled feeding experiments in rabbits confirm that scratching results from high silica content in the monocotyledonous dominated diet (grass), while pitting prevails in the dicotyledonous dominated diet (lucerne). In the case of our study, where the same animal species was used, an influence of differences in dental morphology or chewing mechanisms can be largely excluded as an explanation of these differences. Because of the use of pelleted diets, the observed differences also cannot be contributed to differences in physical growth forms between monocotyledonous and dicotyledonous plants, or different cropping mechanisms required by the animals.

Our findings are consistent with the general assumption underlying the process of 2D microwear formation in ungulates, with browsers having more pits and grazers having more scratches [15], [41], [43], [45], [46], [48], [52]. Our results are partly in accordance with Gomes et al. [13] who found that grazing murids have a high abundance of scratches, while insectivorous and frugivorous murids have a coarser microwear pattern with a higher number of pits. We detected a similar difference between a grass-dominated diet and another (non-grass) diet. Townsend and Croft [16] pointed out that 2D microwear patterns between feeding types are more subtle among cavimorph rodents than reported for ungulates and primates. In contrast, we found microwear patterns (*Np*, number of pits) of grass and lucerne-fed lagomorphs similar to patterns reported in grazing and browsing ungulates [41]. The higher absolute *Ls* value (length of scratch) is related to the smaller in body size in rabbits and hence the size of the molar surface.

The 3D microtexture parameters describe the surface texture pattern of lagomorphs in great detail. The ISO/FDIS parameters quantify the aspects of the basic geometry of texture and can be interpreted as a comprehensive representation of textures and its functional trait [17]. However, because the diets in the feeding experiment did not include seeds or other large and abrasive particles, the frequently stressed hypothesis that seeds are required to cause pit formation is not corroborated in this study. Instead, our results indicate that pits develop without such substrates. In particular, we found that the fewer silica particles are in the diet, the higher the variability of 3D textures. We relate this observation to a generally lower probability of abrasive wear when comminuting dicots such as lucerne or browse.

We found more significant surface texture parameters of the primary and S-L surface compared to the S-F surface. We link this to our very homogeneous sample which consists of domestic white rabbits only, when compared to interspecific datasets using the S-F surface only [17], [19], [34]. It is obvious that the segmentation parameters are prominent on the primary surface, before further filtering processes are applied. Height, hybrid, spatial and function parameters are dependent on the height distribution, which will be less distinct if a regular long wavelength pattern like form and waviness covers the short wavelength roughness signal. Therefore, these parameters become more prominent, after additional filtering processes, on the S-F and S-L surface.

A very interesting and, to our understanding of functional aspects of dental wear, very important question is why low-abrasion wear results in a more variable texture pattern dominated by plateau like structures and less areas of surface lesions. In particular, the biomechanical properties of teeth and diet responsible for the difference in the orientation of surface features (isotropic for high-abrasion diets, anisotropic for low-abrasion diets) remain to be identified experimentally. As our findings demonstrate, anisotropic orientation is associated with a less consistent, more randomly distributed texture pattern. Due to the lower frequency of abrasion on low-abrasion diets, microwear patterns on such diets must represent signals that accumulated over longer periods of time. For example, plateau-like structures may be present in higher proportions on low-abrasion diets because the lower wear rate does not remove them from the surface. “Overwriting”, so to speak, takes longer and is less uniform on low-abrasion diets.

In contrast, processing high-abrasion diets causes a constant “overwriting” of previous signals with a uniform, isotropic pattern. This leads to the interesting hypothesis that those processes that lead to the microwear signal on low-abrasion diets occur on high-abrasion diets as well, yet are not detected because their traces are constantly overwritten. Because grass eating rodents and ungulates often are hypsodont [53], it appears reasonable to assume that this overwriting is associated with increased wear rates. For the same reason, it is plausible to assume that a specific abrasion-dominated texture forms over a shorter period of time (the ‘last supper’ equivalent), whereas the texture on low-abrasion diets (as often found on low-crowned teeth) represents a variety of texture-shaping incidents that are distributed over a longer series of meals.

In classical microwear research variability of scratch and pit counts on fossil teeth has often been used to infer dietary variability such as seasonality in foraging behaviour [54], [55]. The variability signal in our sample of lucerne-fed rabbits, however, cannot be related to dietary variation, because the diet was constant throughout the experiment. These findings therefore caution against equating variation in the microwear signal with dietary variation, and emphasize that variation in the microwear signal might rather be linked to the overall abrasiveness of the diet.

For future studies, it will be particularly intriguing whether the variable pattern created by low-abrasion diets follows specific rules that are not detected yet, but that will allow additional conclusions for dietary reconstructions. For our understanding of the functional process of tooth wear, the exact conditions that lead to texture patterns associated with low-abrasion diets, such as differential forces due to uneven distributions of food between tooth antagonists, or subtle local differences in enamel hardness, remain to be investigated.

## Supporting Information

File S1 **Supporting figures and tables.** Figure S1 Examples of cecotrophs in the stomach of rabbits. Table S1 Groupwise comparison of microwear and 3D texture parameters. Table S2 Descriptive statistics of microwear parameters. Table S3 Descriptive statistics of 3D texture parameters. Table S4 Statistics from WY-tests for the 3D texture parameters. Table S5 Statistics from pair-wise comparison for the microwear and 3D texture parameters. Table S6 Statistics from Cliff tests for the microwear and 3D texture parameters.
(PDF)
