# Supplementary material for: Dietary Abrasiveness Is Associated with Variability of Microwear and Dental Surface Texture in Rabbits
Source: PLoS One. 2013 Feb 6;8(2):e56167. doi: 10.1371/journal.pone.0056167 (PMC3566079; doi:10.1371/journal.pone.0056167)
Supplement: File S1 — Supporting figures and tables. Figure S1 Examples of cecotrophs in the stomach of rabbits. Table S1 Groupwise comparison of microwear and 3D texture parameters. Table S2 Descriptive statistics of microwear parameters. Table S3 Descriptive statistics of 3D texture parameters. Table S4 Statistics from WY-tests for the 3D texture parameters. Table S5 Statistics from pair-wise comparison for the microwear and 3D texture parameters. Table S6 Statistics from Cliff tests for the microwear and 3D texture parameters. (PDF) [file pone.0056167.s001.pdf]

Supporting Information (S1)

Figure S1. Examples of cecotrophs in the stomach of rabbits.

Examples of cecotrophs visible in the stomachs of rabbits at dissection, a) from a teaching course and b) and c) from animals used in this study (scale = 2cm). Note that cecotrophs appear in the stomach intact, indicating that they are not subjected to mastication, i.e. that the practice of coprophagy is unlikely to contribute significantly to overall tooth wear.

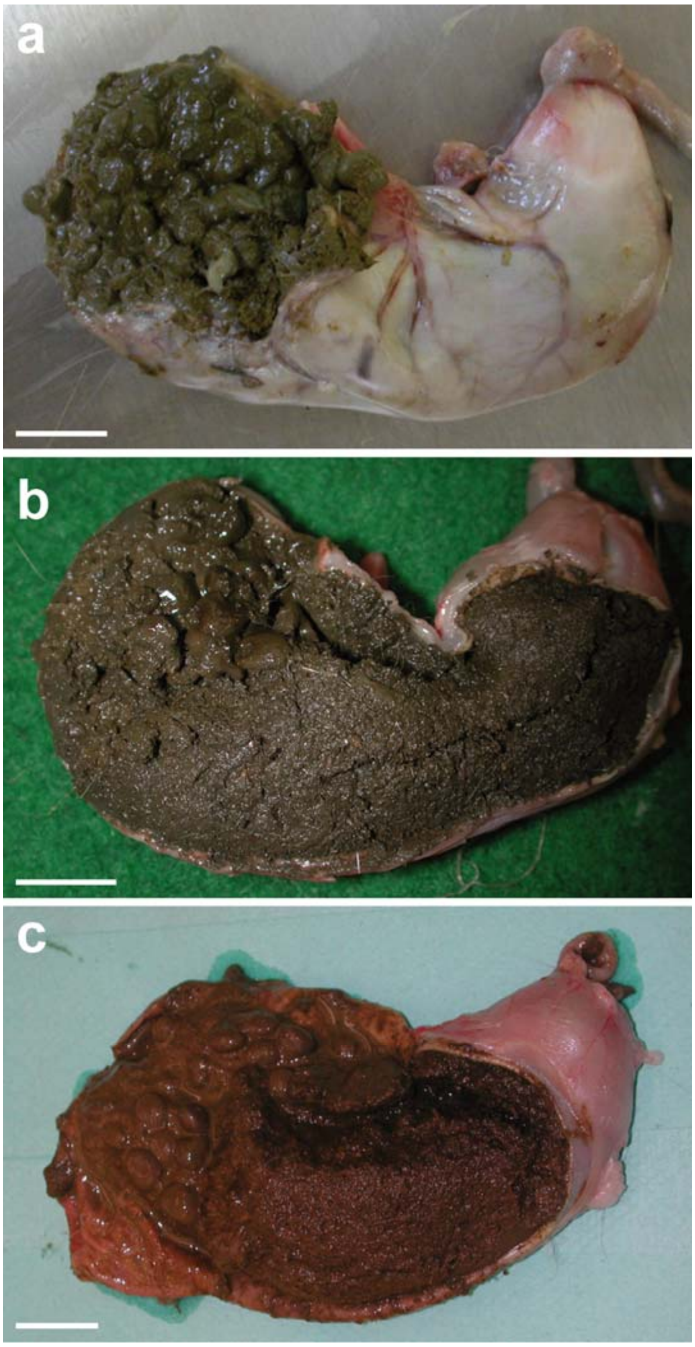

Table S1. Groupwise comparison of microwear and 3D texture parameters.

Groupwise comparison of microwear (*Ls* = length of scratches, *Np* = number of pits, *Np10* = number of the ten largest pits), silica (*SC* [%]) and 3D texture parameters (*Sal* = auto correlation length [µm], *Sda* = closed dale area [μm^2], *Sdr* = developed interfacial area [%], *Sdq* = root mean square gradient, *Sp* = maximum peak height [µm], *Ssk* = skewness, *Sv* = maximum pit height [µm], *Sxp* =extreme peak height [µm], *Sz* = maximum height [µm], *S5v* = five point pit height [µm], *Vvv* = pit void volume [$\mu m^3/\mu m^2$]). Bold lettering = $p \leq 0.05$ for WY/PW/CM-tests, regular = $p \leq 0.05$ in one test only) for the given pair of feeding groups (G = grass meal, GO = grass meal with crushed oats, LO = lucerne with crushed oats, and L = lucerne) for the primary surface (A), S-F surface (B), and S-L surface (C). Statistical analysis in R are applied using the packages xlsReadWrite 1.5.4 (Sutter H.-P., http://CRAN.R-project.org/package=xlsReadWrite), doBy 4.2.3 (Højsgaard S., Wright K., Leidi A. A., http://CRAN.R-project.org/package=doBy), R.utils 1.6.2 (Bengtsson, H., http://CRAN.R-project.org/package=R.utils), RSvgDevice 0.6.4.1 (Luciani, T. J., http://www.darkridge.com/~jake/RSvg/) and WRS 0.12.1 (Wilcox, R. R., Schoenbrodt F., http://R-Forge.R-project.org/projects/wrs/).

**Group 1**<br>**Group 2**	**GO**<br>**G**	**GO**<br>**LO**	**GO**<br>**L**	**G**<br>**LO**	**G**<br>**L**	**LO**<br>**L**
Silica	***SC***	***SC***	***SC***	***SC***	***SC***	***SC***
Microwear	***Ls***, ***Np***, *Np10*	***Ls***, ***Np***	***Ls***, ***Np***	*Np10*	none	none
Texture (A)	***Sda***	***Sda***, *Sdv*	*Sda*	none	none	none
Texture (B)	*Sz, Sdr, Sdq*	none	none	none	none	none
Texture (C)	*S5v, Sal,* ***Sp***, *Ssk, Sdr, Sdq*	*Sz*	*S5v,* ***Sp***, *Ssk, Sv, Sxp, Sz, Vvv*	*Ssk*	*Sal*	none

Table S2. Descriptive statistics of microwear parameters.

Descriptive statistical values (M = mean and SD = standard deviation) given for the feeding groups (G = grass meal, GO = grass meal with crushed oats, LO = lucerne with crushed oats, and L = lucerne). Key to lettering: silica content of 1g animal feed (*S* in mg/g), percentage of silica content (*SC* in %) and the microwear parameters (*Np* = number of pits, *Np10* = number of pits >10µm, *Np5* = number of pits >5µm, *Ns* = number of scratches, *Nws5* = number of scratches wider than 5µm, *Nws10* = number of scratches wider than 10µm, *Ls* = length of scratches), n = number of specimen.

**Group**	**G**		**GO**		**L**		**LO**	
**Parameter**	M	SD	M	SD	M	SD	M	SD
S	11.96	3.24	9.33	2.06	0.93	0.99	0.12	0.23
SC	1.20	0.32	0.93	0.21	0.09	0.10	0.01	0.02
n	10		10		10		10	
Np	21.71	4.72	32.92	9.00	45.86	14.76	39.42	11.72
Np10	0.43	0.53	2.58	1.80	0.43	0.79	2.58	2.40
Np5	4.14	1.82	7.58	3.47	6.36	5.42	7.42	4.52
Ns	8.71	3.60	5.67	1.97	4.50	1.94	4.25	3.27
Nws5	0.93	1.02	1.33	0.52	0.21	0.39	0.17	0.41
Nws10	0.25	0.55	0.14	0.36	0	0	0	0
Ls	59.05	4.78	50.40	6.63	38.94	9.63	48.60	5.93
n	7		6		6		7	

Table S3. Descriptive statistics of 3D texture parameters.

Descriptive statistical values (M = mean and SD = standard deviation) given for the 3D texture parameters (Pa) grouped by the three surface types (A, B, C) and feeding groups (G = grass meal, GO = grass meal with crushed oats, LO = lucerne with crushed oats, and L = lucerne). Key to lettering of the parameters: *Sa* = arithmetical mean height [µm], *Sal* = auto-correlation length (s = 0.2, [µm]), *Sda* = closed dale area [µm2], *Sdq* = root mean square gradient of the scale limited surface, *Sdr* = developed interfacial area ratio of the scale limited surface [%], *Sdv* = closed dale volume [µm3], *Sha* = mean hill area [µm2], *Shv* = closed hill volume [µm3], *Sku* = kurtosis of the scale limited surface, *Smc* = areal material ratio function of the scale limited surface (p = 10%, [µm]), *Smr* = areal material ratio function of the scale limited surface (c = 1 um under the highest peak [%]), *Sp* = maximum peak height [µm], *Spc* = arithmetic mean peak curvature [1/µm], *Spd* = density of peaks [1/µm2], *Sq* = root mean square height of the scale limited surface [µm], *Ssk* = skewness of the scale limited surface, *Std* = texture direction [°], *Str* = texture aspect ratio (s = 0.2), *Sv* = maximum pit height [µm], *Sxp* = peak extreme height difference in height between p% and q% (p = 50%, q =97.5%, [µm]), *Sz* = maximum height of the scale limited surface [µm], *S10z* = ten-point height of the surface [µm], *S5p* = five-point peak height [µm], *S5v* = five-point valley height [µm], *Vm* = material volume at a given height (p = 10% [µm3/µm2]), *Vmc* = material volume of the core [µm3/µm2], *Vmp* = material volume of peaks (p = 10%, [µm3/µm2]), *Vv* = void volume at a given height (p = 10%, [µm3/µm2]), *Vvc* = void volume of the core (p = 10%, q =80% [µm3/µm2]), *Vvv* = void volume of the valley (p = 80% [µm3/µm2]).

	**Primary surface (A)**								**S-F surface (B)**								**S-L surface (C)**							
	**G**		**GO**		**L**		**LO**		**G**		**GO**		**L**		**LO**		**G**		**GO**		**L**		**LO**	
**Pa**	M	SD	M	SD	M	SD	M	SD	M	SD	M	SD	M	SD	M	SD	M	SD	M	SD	M	SD	M	SD
S10z	12.36	2.60	16.98	3.38	14.20	6.67	16.88	5.64	12.53	2.72	18.29	4.87	13.61	6.59	16.91	5.57	12.26	2.53	17.11	3.38	14.39	6.67	16.82	5.49
S5p	10.07	2.09	14.27	2.87	11.38	5.78	13.78	4.83	10.23	2.33	15.35	4.25	10.98	5.77	13.79	4.80	10.16	2.13	14.34	2.95	11.55	5.69	13.77	4.81
S5v	2.29	0.55	2.72	0.53	2.81	1.00	3.10	0.98	2.29	0.53	2.94	0.65	2.63	0.89	3.12	0.95	2.10	0.43	2.77	0.48	2.84	1.09	3.05	0.82
Sa	0.90	0.20	0.87	0.25	1.09	0.37	1.06	0.20	0.54	0.15	0.59	0.17	0.56	0.13	0.59	0.13	0.35	0.10	0.40	0.05	0.42	0.09	0.46	0.09
Sal	25.84	3.61	24.03	5.16	27.25	2.82	25.59	4.08	11.96	4.26	10.17	2.58	11.35	1.45	10.33	0.94	5.13	0.75	4.11	0.83	5.42	1.39	5.05	1.51
Sda	3.85	2.13	2.03	1.29	1.38	0.83	2.15	0.76	2.27	1.59	2.43	0.73	1.35	0.77	1.82	0.73	2.16	1.03	1.46	0.84	1.43	0.59	1.40	0.46
Sdq	0.91	0.28	1.37	0.25	1.05	0.48	1.33	0.65	1.01	0.37	1.53	0.61	0.98	0.45	1.33	0.65	0.91	0.28	1.37	0.25	1.06	0.48	1.33	0.65
Sdr	31.63	16.91	65.33	23.12	46.39	36.27	71.39	66.95	39.06	24.94	88.23	75.71	40.64	34.55	71.30	66.97	31.59	16.72	65.45	23.18	46.56	36.19	71.55	66.82

Sdv	8.2E-08	3.8E-08	4.2E-08	2.6E-08	2.2E-08	1.6E-08	6.2E-08	5.9E-08	5.1E-08	1.8E-08	7.0E-08	3.8E-08	3.6E-08	2.5E-08	5.6E-08	3.6E-08	4.3E-08	1.6E-08	4.1E-08	2.5E-08	3.9E-08	1.1E-08	4.5E-08	1.7E-08
Sha	1.76	1.29	1.10	0.51	1.66	0.47	1.68	1.04	1.54	1.19	1.10	0.49	1.61	0.42	1.58	1.10	1.09	0.91	0.73	0.42	1.07	0.41	1.15	1.00
Shv	3.4E-08	2.4E-08	2.4E-08	1.0E-08	3.8E-08	2.5E-08	5.1E-08	5.8E-08	2.8E-08	1.8E-08	2.9E-08	1.2E-08	4.3E-08	1.9E-08	3.9E-08	2.5E-08	2.0E-08	1.3E-08	1.7E-08	1.2E-08	2.3E-08	6.9E-09	2.7E-08	2.0E-08
Sku	15.26	16.21	16.27	12.47	6.78	6.10	9.62	5.97	44.05	46.07	45.74	23.84	26.16	23.52	32.59	15.62	60.52	43.10	93.94	39.10	49.16	47.01	54.93	27.25
Smc	1.24	0.29	1.25	0.36	1.49	0.43	1.46	0.30	0.79	0.25	0.87	0.27	0.79	0.17	0.84	0.18	0.53	0.17	0.56	0.07	0.62	0.14	0.66	0.13
Smr	0.00	0.00	0.00	0.01	0.00	0.00	0.00	0.00	0.00	0.00	0.00	0.00	0.00	0.01	0.00	0.00	0.00	0.00	0.00	0.00	0.00	0.00	0.00	0.00
Sp	12.60	2.08	17.38	4.17	14.16	6.52	17.24	5.58	14.06	2.76	19.76	4.90	14.98	7.08	18.85	6.24	13.08	2.30	18.87	3.41	16.20	6.67	18.95	6.00
Spc	36.48	8.19	48.88	8.73	42.23	18.54	50.33	15.62	37.30	8.43	50.84	10.61	40.13	19.44	49.40	15.80	35.45	8.07	48.34	9.01	42.39	18.14	49.47	15.86
Spd	0.01	0.00	0.01	0.00	0.01	0.00	0.01	0.01	0.01	0.01	0.01	0.00	0.01	0.00	0.01	0.01	0.01	0.00	0.01	0.00	0.01	0.00	0.01	0.01
Sq	1.17	0.25	1.16	0.26	1.37	0.46	1.39	0.25	0.76	0.17	0.91	0.31	0.79	0.19	0.87	0.24	0.52	0.14	0.65	0.11	0.61	0.14	0.71	0.19
Ssk	-0.14	0.84	0.32	0.84	-0.54	0.52	-0.64	0.87	1.55	1.96	2.14	1.29	0.65	1.56	1.06	1.62	2.66	1.52	4.69	1.36	1.91	2.30	2.60	2.05
Std	55.89	26.84	66.29	22.72	73.94	35.91	86.69	33.17	66.22	28.89	60.99	27.32	80.36	33.83	86.69	33.17	60.55	26.21	66.29	22.72	73.94	35.91	85.53	34.95
Str	0.33	0.06	0.37	0.08	0.37	0.08	0.40	0.13	0.56	0.13	0.57	0.17	0.55	0.14	0.66	0.14	0.67	0.08	0.70	0.10	0.64	0.11	0.67	0.06
Sv	5.95	2.33	6.56	0.93	7.40	2.37	8.32	2.23	4.35	1.39	6.35	2.90	5.39	1.73	6.05	2.21	4.31	1.78	4.85	1.09	6.36	2.04	6.49	2.08
Sxp	2.83	0.93	2.62	0.71	3.45	1.51	3.46	0.57	1.56	0.38	1.67	0.35	1.81	0.40	1.90	0.36	0.99	0.26	1.16	0.11	1.23	0.27	1.35	0.25
Sz	18.54	3.65	23.94	4.30	21.56	8.50	25.57	7.44	18.40	3.39	26.11	7.54	20.37	8.69	24.90	8.11	17.39	3.77	23.71	4.44	22.57	7.59	25.44	7.98
Vm	0.04	0.02	0.04	0.02	0.03	0.01	0.04	0.02	0.04	0.01	0.06	0.03	0.04	0.02	0.04	0.03	0.03	0.01	0.05	0.02	0.04	0.01	0.05	0.02
Vmc	1.01	0.26	0.99	0.33	1.27	0.45	1.20	0.25	0.57	0.18	0.59	0.14	0.58	0.16	0.62	0.12	0.36	0.12	0.39	0.05	0.43	0.11	0.46	0.09
Vmp	0.04	0.02	0.04	0.02	0.03	0.01	0.04	0.02	0.04	0.01	0.06	0.03	0.04	0.02	0.04	0.03	0.03	0.01	0.05	0.02	0.04	0.01	0.05	0.02
Vv	1.27	0.28	1.29	0.35	1.52	0.42	1.50	0.31	0.83	0.25	0.93	0.30	0.84	0.19	0.89	0.20	0.56	0.17	0.61	0.08	0.66	0.14	0.70	0.14
Vvc	1.10	0.25	1.13	0.33	1.32	0.35	1.28	0.30	0.73	0.24	0.81	0.26	0.72	0.17	0.76	0.18	0.49	0.16	0.53	0.08	0.58	0.13	0.61	0.13
Vvv	0.17	0.06	0.16	0.03	0.20	0.08	0.22	0.04	0.10	0.02	0.12	0.03	0.12	0.02	0.12	0.03	0.06	0.02	0.08	0.01	0.08	0.02	0.09	0.02
n	7		7		7		8		7		7		7		8		7		7		7		8	

Table S4. Statistics from WY-tests for the 3D texture parameters.

Test statistics from WY-tests with 15% trimming for the 3D texture analyses of the primary surface (A), S-F surface (B), and S-L surface (C). Values in bold indicate a significant difference ($p \leq 0.05$). *Ft* = test statistics, nu1 and nu2 = 1st and 2nd degree of freedom, respectively, *p* = significance level. For key to parameter lettering see App. 2.

	**(A)**				**(B)**				**(C)**			
**Parameter**	**Ft**	***p***	**nu1**	**nu2**	**Ft**	***p***	**nu1**	**nu2**	**Ft**	***p***	**nu1**	**nu2**
S10z	3.040	0.086	3	8.835	2.782	0.103	3	8.852	3.310	0.073	3	8.772
S5p	3.145	0.081	3	8.739	2.538	0.122	3	8.998	2.861	0.099	3	8.725
S5v	1.544	0.269	3	9.089	1.837	0.209	3	9.271	5.474	**0.021**	3	8.868
Sa	2.411	0.135	3	8.920	0.177	0.910	3	9.324	0.865	0.495	3	8.799
Sal	0.942	0.459	3	9.215	0.806	0.525	3	8.148	4.445	**0.035**	3	9.043
Sda	4.359	**0.037**	3	8.971	2.332	0.141	3	9.175	0.537	0.669	3	8.704
Sdq	1.863	0.204	3	9.259	1.515	0.274	3	9.324	1.853	0.206	3	9.261
Sdr	1.868	0.205	3	9.064	1.344	0.318	3	9.409	1.865	0.205	3	9.053
Sha	1.114	0.393	3	9.028	1.342	0.322	3	8.760	0.670	0.591	3	9.083
Sku	2.606	0.129	3	7.438	0.744	0.553	3	8.722	3.181	0.081	3	8.533
Smc	2.047	0.179	3	8.884	0.110	0.952	3	9.303	0.803	0.525	3	8.508
Smr	0.655	0.601	3	8.266	0.659	0.598	3	8.777	2.426	0.137	3	8.425
Sp	3.536	0.071	3	7.618	3.449	0.072	3	7.990	4.192	**0.043**	3	8.629
Spc	2.731	0.110	3	8.490	2.065	0.178	3	8.657	2.896	0.097	3	8.671
Spd	1.124	0.389	3	9.159	1.639	0.250	3	8.739	1.358	0.318	3	8.718
Sq	2.004	0.187	3	8.626	0.154	0.925	3	9.321	0.878	0.488	3	9.010
Ssk	2.866	0.106	3	7.787	0.788	0.529	3	9.264	4.498	**0.034**	3	9.169
Std	1.040	0.421	3	9.063	0.625	0.617	3	8.985	0.708	0.575	3	7.566
Str	0.558	0.657	3	8.542	1.629	0.251	3	8.900	0.329	0.805	3	8.736
Sv	1.084	0.409	3	8.029	1.067	0.409	3	9.360	3.862	**0.048**	3	9.424
Sxp	2.297	0.145	3	9.166	1.101	0.398	3	9.049	1.793	0.222	3	8.575
Sz	2.284	0.149	3	8.784	2.972	**0.095**	3	8.213	4.282	**0.042**	3	8.432
Vm	2.093	0.172	3	8.859	0.251	0.858	3	8.988	1.178	0.371	3	9.092
Vmc	2.199	0.158	3	8.956	0.209	0.887	3	9.059	1.347	0.325	3	8.168
Vmp	2.093	0.172	3	8.859	0.251	0.858	3	8.988	1.178	0.371	3	9.092
Vv	1.950	0.193	3	8.898	0.089	0.964	3	9.324	0.758	0.547	3	8.564
Vvc	1.779	0.221	3	8.926	0.049	0.985	3	9.248	0.673	0.591	3	8.434
Vvv	2.834	0.098	3	9.112	1.444	0.293	3	9.149	2.487	0.128	3	8.869
Sdv	5.971	**0.016**	3	9.128	0.762	0.545	3	8.525	0.162	0.920	3	9.104
Shv	0.795	0.529	3	8.384	1.139	0.382	3	9.437	1.209	0.366	3	8.248

Table S5. Statistics from pair-wise comparison for the microwear and 3D texture parameters.

Test statistics from heteroscedastic pair-wise comparison test (analog to Dunnett's T3 test) with 15% trimming for the microwear/SC = silica (a) and 3D texture (b) parameters of the primary surface (A), S-F surface (B), and S-L surface (C) grouped by the feeding groups (G = grass meal, GO = grass meal with crushed oats, LO = lucerne with crushed oats, and L = lucerne). Values in bold indicate a significant difference ($p \leq 0.05$). t = test statistics, df = degree of freedom, *p* = significance level. For key to parameter lettering see Apps. 1 and 2.

a)

Parameter	Group 1	Group2	t	df	*p*
Ls	GO	G	2.612	8.49	**0.030**
	GO	LO	3.785	5.16	**0.012**
	GO	L	3.397	8.82	**0.008**
	G	LO	1.997	6.38	0.090
	G	L	0.495	9.88	0.631
	LO	L	1.714	5.94	0.138
Np	GO	G	2.753	7.25	**0.027**
	GO	LO	4.289	4.85	**0.008**
	GO	L	3.476	6.41	**0.012**
	G	LO	2.095	7.10	0.074
	G	L	1.077	9.38	0.308
	LO	L	1.027	8.37	0.333
Np10	GO	G	2.760	6.51	**0.030**
	GO	LO	0.505	7.93	0.628
	GO	L	2.138	5.88	0.077
	G	LO	3.047	6.27	**0.021**
	G	L	0.000	9.28	1.000
	LO	L	2.350	5.73	0.059
Np5	GO	G	1.992	7.41	0.084
	GO	LO	0.558	4.65	0.603
	GO	L	1.517	6.52	0.176
	G	LO	0.571	6.31	0.588
	G	L	0.072	9.37	0.944
	LO	L	0.478	7.55	0.646
Ns	GO	G	1.548	5.38	0.178
	GO	LO	2.044	5.86	0.088
	GO	L	1.983	7.39	0.086
	G	LO	0.933	8.31	0.377
	G	L	0.910	8.20	0.389
	LO	L	0.152	8.62	0.883
SC	GO	G	3.892	46.13	**<0.001**
	GO	LO	19.944	29.28	**<0.001**
	GO	L	18.010	33.08	**<0.001**
	G	LO	27.988	40.25	**<0.001**
	G	L	23.206	56.05	**<0.001**
	LO	L	4.985	44.34	**<0.001**

b)

			**(A)**			**(B)**			**(C)**		
			**t**	**df**	***p***	**t**	**df**	***p***	**t**	**df**	***p***
S10z	GO	G	3.068	7.14	0.018	2.914	6.94	0.023	3.182	6.88	0.016
	GO	LO	0.503	4.71	0.638	0.185	4.86	0.860	0.614	4.67	0.568
	GO	L	1.611	6.23	0.157	1.537	6.43	0.172	1.704	6.20	0.138

	G	LO	0.990	5.42	0.364	1.384	5.87	0.217	0.962	5.53	0.376
	G	L	0.186	7.35	0.858	0.342	7.86	0.741	0.218	7.54	0.833
	LO	L	0.693	8.29	0.507	0.920	8.38	0.383	0.655	8.24	0.530
S5p	GO	G	3.121	6.82	0.017	2.798	7.40	0.025	2.963	6.65	0.022
	GO	LO	0.379	4.60	0.722	0.106	5.03	0.920	0.406	4.65	0.702
	GO	L	1.594	6.18	0.161	1.450	6.92	0.191	1.563	6.26	0.167
	G	LO	1.102	5.43	0.317	1.419	5.77	0.208	1.084	5.65	0.322
	G	L	0.290	7.57	0.780	0.461	8.03	0.657	0.326	7.87	0.753
	LO	L	0.737	8.03	0.482	0.901	8.05	0.394	0.702	8.07	0.502
S5v	GO	G	1.938	7.95	0.089	2.051	7.70	0.076	3.601	7.43	**0.008**
	GO	LO	1.050	5.42	0.338	0.770	6.57	0.468	1.577	4.54	0.181
	GO	L	1.748	7.24	0.123	1.950	7.08	0.092	2.994	6.47	**0.022**
	G	LO	0.012	5.22	0.991	0.849	7.35	0.423	0.071	4.94	0.946
	G	L	0.578	6.96	0.581	0.532	7.84	0.609	0.765	7.41	0.468
	LO	L	0.405	8.41	0.695	1.137	8.87	0.285	0.413	7.16	0.692
Sa	GO	G	1.107	6.31	0.309	0.232	7.46	0.823	0.662	5.48	0.535
	GO	LO	0.740	7.27	0.483	0.222	7.76	0.830	0.774	7.99	0.461
	GO	L	1.183	8.84	0.267	0.720	8.42	0.491	1.479	6.29	0.187
	G	LO	1.693	5.29	0.148	0.001	7.93	0.999	0.349	5.38	0.740
	G	L	2.591	7.80	0.033	0.573	9.00	0.580	1.375	8.90	0.203
	LO	L	0.230	7.48	0.825	0.550	8.94	0.596	0.485	6.14	0.645
Sal	GO	G	0.356	7.65	0.732	0.344	6.93	0.741	3.389	7.93	**0.010**
	GO	LO	1.028	6.41	0.341	0.237	4.98	0.822	0.792	4.93	0.465
	GO	L	0.017	9.00	0.987	0.424	4.40	0.692	0.267	6.98	0.797
	G	LO	1.658	7.26	0.140	0.887	6.12	0.409	2.401	5.11	0.060
	G	L	0.349	8.61	0.736	0.031	4.92	0.976	2.343	7.31	**0.050**
	LO	L	0.917	7.35	0.388	1.639	6.95	0.146	0.498	7.73	0.632
Sda	GO	G	2.362	6.81	**0.051**	1.449	6.71	0.192	0.877	7.92	0.406
	GO	LO	3.561	7.59	**0.008**	1.656	8.00	0.136	1.164	7.45	0.280
	GO	L	2.771	8.33	**0.023**	0.087	8.82	0.932	1.324	6.12	0.233
	G	LO	0.677	7.67	0.518	2.689	6.74	0.032	0.115	7.08	0.911
	G	L	0.428	6.24	0.683	1.397	6.74	0.207	0.154	5.77	0.883
	LO	L	1.396	7.19	0.204	1.769	8.80	0.112	0.037	7.39	0.971
Sdq	GO	G	2.454	8.00	0.040	1.836	7.67	0.105	2.448	8.00	0.040
	GO	LO	0.426	6.34	0.684	0.244	7.73	0.814	0.434	6.35	0.679
	GO	L	1.253	8.36	0.244	0.874	8.92	0.405	1.256	8.34	0.243
	G	LO	1.286	6.31	0.244	1.886	6.99	0.101	1.279	6.34	0.246
	G	L	0.664	8.33	0.525	0.665	8.33	0.524	0.653	8.33	0.531
	LO	L	0.605	8.39	0.561	1.025	8.92	0.332	0.604	8.43	0.562
Sdr	GO	G	2.377	6.73	0.051	1.770	7.74	0.116	2.371	6.69	0.051
	GO	LO	0.639	5.89	0.547	0.176	7.83	0.865	0.648	5.88	0.542
	GO	L	1.413	7.18	0.200	1.002	8.36	0.344	1.415	7.14	0.199
	G	LO	1.192	7.63	0.269	1.818	7.99	0.107	1.184	7.65	0.272
	G	L	0.490	8.85	0.636	0.499	8.90	0.630	0.478	8.85	0.644
	LO	L	0.624	8.93	0.548	1.095	8.82	0.302	0.623	8.94	0.549
Sdv	GO	G	1.991	7.75	0.083	0.965	5.62	0.374	0.225	6.29	0.829
	GO	LO	4.437	6.80	**0.003**	1.057	6.22	0.330	0.532	7.46	0.610
	GO	L	1.822	8.99	0.102	0.341	7.89	0.742	0.130	8.94	0.899
	G	LO	1.588	6.10	0.163	1.589	7.76	0.152	0.107	5.41	0.918
	G	L	0.256	8.63	0.804	1.189	4.99	0.288	0.314	6.52	0.763

	LO	L	2.036	7.87	0.077	0.895	5.41	0.409	0.669	8.71	0.521
Sha	GO	G	0.879	6.42	0.411	0.506	6.30	0.630	0.504	6.60	0.631
	GO	LO	0.340	5.37	0.747	0.667	4.71	0.536	0.475	5.46	0.653
	GO	L	0.062	5.83	0.953	0.004	6.08	0.997	0.062	6.58	0.953
	G	LO	1.935	7.29	0.093	2.003	6.09	0.091	1.483	7.24	0.180
	G	L	1.438	8.28	0.187	0.753	8.67	0.471	0.616	8.80	0.554
	LO	L	0.469	8.99	0.650	1.172	7.80	0.276	0.810	8.73	0.439
Shv	GO	G	0.800	6.47	0.452	0.667	7.96	0.524	0.522	7.72	0.617
	GO	LO	0.257	7.74	0.804	1.895	7.96	0.095	0.950	4.70	0.388
	GO	L	0.192	5.25	0.855	1.050	8.71	0.322	0.597	8.77	0.566
	G	LO	0.992	5.80	0.361	1.277	8.00	0.237	1.901	5.02	0.115
	G	L	1.599	7.26	0.152	0.521	8.48	0.616	1.192	8.99	0.264
	LO	L	0.155	4.87	0.883	0.504	8.49	0.627	0.169	5.96	0.872
Sku	GO	G	0.289	6.40	0.782	0.189	4.73	0.858	1.871	5.15	0.119
	GO	LO	1.038	4.05	0.357	0.597	5.71	0.574	0.501	7.48	0.631
	GO	L	0.367	5.11	0.729	0.225	4.63	0.831	0.038	7.33	0.971
	G	LO	2.359	4.16	0.075	1.524	6.79	0.173	2.071	4.68	0.097
	G	L	1.054	7.05	0.326	1.044	8.70	0.325	2.500	7.70	0.038
	LO	L	1.706	5.46	0.144	0.774	6.64	0.466	0.590	6.10	0.577
Smc	GO	G	0.776	6.11	0.467	0.218	7.85	0.833	0.447	5.25	0.673
	GO	LO	0.901	7.49	0.396	0.227	7.88	0.826	0.725	7.77	0.490
	GO	L	1.196	8.71	0.263	0.590	8.35	0.571	1.150	4.87	0.303
	G	LO	1.674	5.30	0.152	0.011	8.00	0.991	0.500	5.72	0.636
	G	L	2.341	7.77	0.048	0.398	8.82	0.700	1.410	8.23	0.195
	LO	L	0.095	7.55	0.927	0.385	8.79	0.710	0.292	5.22	0.781
Smr	GO	G	0.907	5.52	0.402	1.312	5.55	0.241	0.165	4.44	0.876
	GO	LO	1.295	4.30	0.260	1.416	4.58	0.221	2.862	5.78	0.030
	GO	L	0.783	6.62	0.461	0.952	6.32	0.376	1.914	8.99	0.088
	G	LO	0.676	5.47	0.527	0.054	6.56	0.958	0.576	4.10	0.595
	G	L	0.113	8.76	0.913	0.497	8.93	0.631	0.479	4.52	0.654
	LO	L	0.661	6.05	0.533	0.622	7.18	0.553	0.306	6.86	0.769
Sp	GO	G	2.736	4.43	0.047	2.992	5.05	0.030	3.297	6.29	**0.015**
	GO	LO	0.517	4.15	0.631	0.253	4.24	0.812	0.939	4.58	0.395
	GO	L	2.319	5.40	0.064	2.003	5.59	0.096	2.401	6.31	**0.051**
	G	LO	0.997	6.55	0.354	1.130	5.70	0.304	0.764	5.76	0.475
	G	L	0.065	9.00	0.949	0.052	8.37	0.960	0.056	8.33	0.957
	LO	L	0.910	7.36	0.392	0.983	7.52	0.356	0.724	7.66	0.491
Spc	GO	G	2.641	6.00	0.039	2.413	6.37	0.050	2.774	6.41	0.030
	GO	LO	0.514	4.44	0.632	0.081	4.54	0.939	0.699	4.60	0.518
	GO	L	1.955	6.15	0.097	1.532	6.47	0.173	1.977	6.36	0.093
	G	LO	0.747	5.58	0.485	1.076	5.59	0.326	0.723	5.72	0.498
	G	L	0.069	8.37	0.947	0.215	8.47	0.834	0.021	8.28	0.984
	LO	L	0.725	7.30	0.491	0.841	7.19	0.428	0.661	7.66	0.528
Spd	GO	G	0.934	7.97	0.378	0.694	7.71	0.508	0.637	7.82	0.542
	GO	LO	0.791	6.91	0.455	1.018	5.06	0.355	1.125	6.22	0.302
	GO	L	0.268	7.53	0.796	0.252	6.74	0.809	0.480	5.50	0.650
	G	LO	1.947	7.17	0.092	2.201	5.54	0.074	2.081	6.81	0.077
	G	L	1.368	7.86	0.209	1.169	7.69	0.277	1.415	6.00	0.207
	LO	L	0.638	8.99	0.539	1.116	8.01	0.297	1.083	8.06	0.310

Sq	GO	G	0.858	5.75	0.425	0.464	6.88	0.657	1.532	6.69	0.171
	GO	LO	0.635	7.09	0.545	0.034	7.57	0.973	0.854	7.54	0.419
	GO	L	1.295	9.00	0.228	0.526	8.98	0.612	1.454	8.45	0.182
	G	LO	1.353	4.86	0.236	0.500	7.74	0.631	0.289	5.77	0.782
	G	L	2.387	6.75	0.050	0.172	8.04	0.868	0.488	6.69	0.641
	LO	L	0.444	7.88	0.669	0.548	8.70	0.598	0.610	9.00	0.557
Ssk	GO	G	0.709	6.81	0.502	1.062	7.61	0.321	2.443	6.94	**0.045**
	GO	LO	1.123	4.20	0.322	0.662	7.50	0.528	0.945	7.79	0.373
	GO	L	0.990	6.29	0.359	0.304	8.98	0.768	0.165	8.81	0.872
	G	LO	2.999	4.48	0.035	1.543	7.99	0.162	3.249	6.30	**0.016**
	G	L	2.341	8.38	0.046	1.302	8.37	0.228	2.697	8.46	**0.026**
	LO	L	0.015	5.82	0.989	0.381	8.23	0.713	0.808	8.22	0.442
Std	GO	G	0.602	6.93	0.566	0.942	6.21	0.381	0.067	4.36	0.949
	GO	LO	0.679	5.60	0.524	0.357	5.80	0.734	0.291	4.17	0.785
	GO	L	1.865	7.63	0.101	0.808	7.59	0.444	1.560	5.31	0.176
	G	LO	0.203	7.12	0.845	1.011	7.89	0.342	0.203	7.12	0.845
	G	L	1.171	8.96	0.272	1.411	8.90	0.192	1.171	8.96	0.272
	LO	L	0.783	8.26	0.455	0.317	8.56	0.759	0.783	8.26	0.455
Str	GO	G	1.032	6.46	0.339	0.800	7.77	0.448	0.660	7.27	0.529
	GO	LO	0.983	5.80	0.365	0.297	5.60	0.777	0.417	6.79	0.689
	GO	L	1.201	8.90	0.261	1.423	8.92	0.189	0.121	6.55	0.908
	G	LO	0.535	4.65	0.618	1.434	6.17	0.200	0.921	5.61	0.395
	G	L	0.057	7.67	0.956	0.746	8.95	0.475	0.729	8.13	0.486
	LO	L	0.617	6.45	0.558	2.116	6.88	0.073	0.547	5.09	0.607
Sv	GO	G	0.301	4.59	0.777	1.560	7.94	0.158	1.415	7.99	0.195
	GO	LO	1.010	7.87	0.343	1.010	7.48	0.344	3.144	7.86	**0.014**
	GO	L	1.426	9.00	0.188	1.617	8.12	0.144	2.639	7.33	**0.032**
	G	LO	1.121	4.76	0.315	0.284	7.17	0.785	1.789	7.90	0.112
	G	L	1.595	5.61	0.165	0.505	7.79	0.627	1.706	7.43	0.129
	LO	L	0.550	8.89	0.596	0.671	8.93	0.519	0.447	7.87	0.667
Sxp	GO	G	0.981	7.34	0.358	0.152	6.11	0.884	1.460	5.52	0.199
	GO	LO	0.466	7.99	0.654	1.102	7.97	0.303	1.060	6.53	0.327
	GO	L	1.346	7.77	0.216	1.296	7.47	0.233	2.454	8.24	0.039
	G	LO	1.485	7.19	0.180	1.238	6.32	0.260	0.271	4.56	0.798
	G	L	2.768	8.92	0.022	1.599	8.78	0.145	1.690	7.53	0.132
	LO	L	0.787	7.58	0.455	0.059	7.76	0.954	0.596	5.90	0.573
Sz	GO	G	2.225	6.60	0.064	2.868	5.53	**0.031**	3.108	5.84	0.022
	GO	LO	0.696	4.78	0.519	0.328	4.33	0.758	1.517	4.57	0.195
	GO	L	2.112	6.63	0.075	1.734	5.59	0.137	2.389	5.80	**0.056**
	G	LO	0.543	5.98	0.607	1.004	5.61	0.357	0.270	6.16	0.796
	G	L	0.419	8.45	0.685	0.053	7.61	0.959	0.414	7.82	0.690
	LO	L	0.793	7.81	0.451	0.887	8.29	0.400	0.551	8.72	0.596
Vm	GO	G	1.238	5.49	0.266	0.882	6.49	0.409	1.736	6.37	0.130
	GO	LO	0.709	7.40	0.500	0.002	7.21	0.998	0.738	6.29	0.487
	GO	L	0.286	8.66	0.781	0.136	8.68	0.895	1.403	7.72	0.199
	G	LO	2.584	6.47	0.039	0.786	7.75	0.455	0.796	8.00	0.449
	G	L	0.599	5.99	0.571	0.804	6.29	0.451	0.258	8.91	0.802
	LO	L	0.877	7.56	0.408	0.107	7.13	0.917	0.535	8.88	0.606
Vmc	GO	G	0.985	6.62	0.359	0.089	8.00	0.932	0.403	5.16	0.703

	GO	LO	0.751	6.70	0.478	0.040	7.99	0.969	0.696	7.94	0.506
	GO	L	1.197	8.53	0.263	0.683	7.55	0.515	1.277	4.54	0.263
	G	LO	1.499	5.13	0.193	0.046	7.98	0.964	0.515	5.37	0.627
	G	L	2.562	8.49	0.032	0.587	7.62	0.574	1.912	7.23	0.096
	LO	L	0.097	6.33	0.926	0.619	7.35	0.555	0.394	4.64	0.711
Vmp	GO	G	1.238	5.49	0.266	0.882	6.49	0.409	1.736	6.37	0.130
	GO	LO	0.709	7.40	0.500	0.002	7.21	0.998	0.738	6.29	0.487
	GO	L	0.286	8.66	0.781	0.136	8.68	0.895	1.403	7.72	0.199
	G	LO	2.584	6.47	0.039	0.786	7.75	0.455	0.796	8.00	0.449
	G	L	0.599	5.99	0.571	0.804	6.29	0.451	0.258	8.91	0.802
	LO	L	0.877	7.56	0.408	0.107	7.13	0.917	0.535	8.88	0.606
Vv	GO	G	0.684	6.08	0.519	0.242	7.71	0.815	0.562	5.63	0.596
	GO	LO	0.912	7.62	0.390	0.180	7.79	0.862	0.756	7.82	0.472
	GO	L	1.206	8.61	0.260	0.529	8.42	0.610	1.273	5.07	0.258
	G	LO	1.642	5.38	0.157	0.065	7.99	0.950	0.387	6.13	0.712
	G	L	2.290	7.86	0.052	0.324	8.96	0.753	1.252	8.06	0.246
	LO	L	0.106	7.60	0.919	0.383	8.92	0.711	0.386	5.44	0.714
Vvc	GO	G	0.431	6.13	0.682	0.178	7.87	0.864	0.482	5.65	0.648
	GO	LO	1.095	7.64	0.307	0.001	7.86	0.999	0.717	7.59	0.495
	GO	L	1.278	8.49	0.235	0.324	8.00	0.754	1.152	4.85	0.303
	G	LO	1.649	5.44	0.155	0.192	8.00	0.853	0.416	6.48	0.691
	G	L	2.128	8.05	0.066	0.149	8.56	0.885	1.196	7.46	0.268
	LO	L	0.033	7.47	0.974	0.352	8.57	0.733	0.350	5.36	0.740
Vvv	GO	G	1.014	6.69	0.346	0.588	6.65	0.576	2.168	6.89	0.067
	GO	LO	0.369	7.80	0.722	1.627	7.97	0.142	1.269	6.01	0.251
	GO	L	1.673	8.80	0.129	1.753	8.99	0.114	2.715	8.19	0.026
	G	LO	1.325	6.07	0.233	1.372	6.42	0.216	0.081	4.91	0.938
	G	L	3.071	8.26	0.015	1.521	7.73	0.168	1.450	6.63	0.193
	LO	L	1.152	8.19	0.282	0.144	8.93	0.889	0.925	8.19	0.381

Table S6. Statistics from Cliff tests for the microwear and 3D texture parameters.
Test statistics from Cliff tests of the microwear/silica concnentration (a) and 3D texture (b) parameters grouped by the feeding groups (G = grass meal, GO = grass meal with crushed oats, LO = lucerne with crushed oats, and L = lucerne). Values in bold indicate a significant difference ($p \leq$ pc ≤ 0.05) for a given pair of feeding groups, ph = test statistics, pl = lower 95% confidence interval of ph, pu = upper 95% confidence interval of ph, *p* = significance level (not adjusted for family-wise error), *pc* = critical significance level (adjusted for family-wise error). For key to parameter lettering see Apps. 1 and 2.

a)

Parameter	Group 1	Group2	ph	pl	pu	*p*	pc
Ls	GO	G	0.119	0.024	0.429	0.018	0.0125
	**GO**	**LO**	0.000	0.000	0.348	**0.008**	**0.0083**
	GO	L	0.095	0.017	0.395	0.01	0.01
	G	LO	0.143	0.030	0.471	0.034	0.0167
	G	L	0.361	0.115	0.711	0.48	0.05
	LO	L	0.762	0.426	0.933	0.14	0.025
Np	GO	G	0.845	0.509	0.966	0.045	0.0125
	**GO**	**LO**	0.929	0.639	0.990	**0.006**	**0.01**
	**GO**	**L**	0.976	0.763	0.998	**0.0003**	**0.0083**
	G	LO	0.810	0.472	0.953	0.073	0.0167
	G	L	0.611	0.275	0.867	0.57	0.05
	LO	L	0.357	0.122	0.689	0.44	0.025
Np10	GO	G	0.881	0.519	0.981	0.04	0.0125
	GO	LO	0.459	0.225	0.713	0.78	0.025
	GO	L	0.845	0.515	0.966	0.041	0.0167
	G	LO	0.143	0.031	0.461	0.029	0.01
	G	L	0.458	0.173	0.774	0.83	0.05
	LO	L	0.857	0.538	0.969	0.029	0.0083
Np5	GO	G	0.869	0.537	0.974	0.03	0.0083
	GO	LO	0.541	0.236	0.818	0.83	0.025
	GO	L	0.821	0.434	0.965	0.11	0.01
	G	LO	0.369	0.123	0.709	0.5	0.0125
	G	L	0.514	0.218	0.801	0.94	0.05
	LO	L	0.560	0.245	0.833	0.76	0.0167
Ns	GO	G	0.262	0.077	0.602	0.18	0.0125
	GO	LO	0.184	0.045	0.516	0.062	0.01
	GO	L	0.119	0.024	0.429	0.018	0.0083
	G	LO	0.393	0.148	0.707	0.55	0.025
	G	L	0.292	0.076	0.673	0.32	0.0167
	LO	L	0.464	0.177	0.778	0.86	0.05
SC	GO	G	0.212	0.119	0.348	**<0.0001**	**0.05**
	GO	LO	0	0	0.095	**<0.0001**	**0.025**
	GO	L	0.007	0.001	0.042	**<0.0001**	**0.0167**
	G	LO	0	0	0.072	**<0.0001**	**0.0125**
	G	L	0	0	0.072	**<0.0001**	**0.01**

	LO	L	0.777	0.658	0.863	**<0.0001**	**0.0083**

b)

	Groups		(A) ph	pl	pu	*p*	pc	(B) ph	pl	pu	*p*	pc	(C) ph	pl	pu	*p*	pc
S10z	GO	G	0.88	0.58	0.97	0.02	0.01	0.76	0.41	0.93	0.16	0.01	0.88	0.58	0.97	0.02	0.01
	GO	LO	0.55	0.23	0.84	0.80	0.03	0.59	0.27	0.85	0.63	0.03	0.57	0.24	0.85	0.73	0.03
	GO	L	0.75	0.44	0.92	0.12	0.01	0.79	0.46	0.94	0.09	0.01	0.75	0.44	0.92	0.13	0.01
	G	LO	0.39	0.14	0.71	0.53	0.02	0.47	0.19	0.77	0.87	0.05	0.37	0.13	0.69	0.46	0.02
	G	L	0.46	0.20	0.76	0.84	0.05	0.64	0.33	0.87	0.40	0.01	0.48	0.21	0.77	0.92	0.05
	LO	L	0.66	0.35	0.88	0.34	0.01	0.59	0.29	0.83	0.60	0.02	0.64	0.33	0.87	0.40	0.01
S5p	GO	G	0.88	0.58	0.97	0.02	0.01	0.78	0.42	0.94	0.14	0.01	0.88	0.58	0.97	0.02	0.01
	GO	LO	0.53	0.22	0.82	0.88	0.03	0.59	0.27	0.85	0.63	0.01	0.53	0.22	0.82	0.88	0.05
	GO	L	0.70	0.39	0.89	0.23	0.01	0.75	0.42	0.92	0.14	0.01	0.70	0.39	0.89	0.23	0.01
	G	LO	0.39	0.14	0.71	0.55	0.02	0.49	0.20	0.78	0.96	0.05	0.39	0.14	0.71	0.53	0.02
	G	L	0.48	0.21	0.77	0.92	0.05	0.55	0.26	0.81	0.76	0.03	0.46	0.20	0.76	0.84	0.03
	LO	L	0.66	0.35	0.88	0.34	0.01	0.57	0.27	0.82	0.68	0.02	0.64	0.33	0.87	0.40	0.01
S5v	GO	G	0.73	0.40	0.92	0.19	0.01	0.57	0.26	0.84	0.70	0.05	0.86	0.56	0.97	0.02	0.01
	GO	LO	0.65	0.33	0.88	0.39	0.01	0.61	0.30	0.85	0.52	0.02	0.73	0.39	0.92	0.19	0.01
	GO	L	0.77	0.45	0.93	0.11	0.01	0.77	0.46	0.93	0.09	0.01	0.84	0.53	0.96	0.04	0.01
	G	LO	0.51	0.22	0.79	0.96	0.05	0.57	0.27	0.83	0.69	0.03	0.51	0.22	0.80	0.96	0.05
	G	L	0.57	0.28	0.82	0.67	0.02	0.73	0.42	0.91	0.15	0.01	0.59	0.29	0.83	0.60	0.02
	LO	L	0.57	0.28	0.82	0.68	0.03	0.66	0.34	0.88	0.35	0.01	0.55	0.26	0.82	0.76	0.03
Sa	GO	G	0.39	0.14	0.71	0.55	0.02	0.45	0.18	0.75	0.78	0.05	0.59	0.28	0.84	0.60	0.02
	GO	LO	0.59	0.28	0.84	0.60	0.03	0.67	0.36	0.88	0.31	0.02	0.69	0.37	0.90	0.26	0.01
	GO	L	0.71	0.40	0.90	0.19	0.01	0.79	0.47	0.94	0.08	0.01	0.79	0.48	0.94	0.07	0.01
	G	LO	0.71	0.37	0.91	0.24	0.01	0.78	0.44	0.94	0.12	0.01	0.57	0.27	0.83	0.69	0.05
	G	L	0.73	0.40	0.92	0.19	0.01	0.71	0.38	0.91	0.23	0.01	0.68	0.37	0.88	0.27	0.01
	LO	L	0.54	0.24	0.81	0.84	0.05	0.55	0.25	0.82	0.77	0.03	0.57	0.27	0.82	0.68	0.03
Sal	GO	G	0.43	0.17	0.73	0.68	0.03	0.16	0.04	0.51	0.06	0.01	0.17	0.04	0.50	0.05	0.01
	GO	LO	0.61	0.29	0.86	0.53	0.02	0.57	0.26	0.84	0.70	0.05	0.63	0.30	0.88	0.49	0.02
	GO	L	0.46	0.20	0.75	0.84	0.05	0.29	0.09	0.61	0.22	0.01	0.54	0.25	0.81	0.80	0.05
	G	LO	0.68	0.36	0.89	0.29	0.01	0.76	0.42	0.93	0.14	0.01	0.78	0.44	0.94	0.12	0.01
	G	L	0.61	0.31	0.84	0.52	0.01	0.68	0.37	0.89	0.29	0.02	0.75	0.43	0.92	0.13	0.01
	LO	L	0.38	0.14	0.69	0.47	0.01	0.36	0.13	0.67	0.40	0.03	0.42	0.17	0.72	0.65	0.03
Sda	GO	G	0.20	0.05	0.54	0.08	0.02	0.37	0.13	0.69	0.46	0.02	0.27	0.08	0.60	0.18	0.01
	GO	LO	0.04	0.00	0.28	**0.00**	0.01	0.16	0.04	0.51	0.06	0.01	0.24	0.07	0.58	0.14	0.01
	GO	L	0.18	0.05	0.49	0.04	0.01	0.39	0.15	0.70	0.53	0.05	0.23	0.07	0.54	0.09	0.01
	G	LO	0.35	0.12	0.67	0.39	0.03	0.33	0.11	0.65	0.32	0.01	0.49	0.21	0.78	0.96	0.03
	G	L	0.57	0.26	0.83	0.69	0.05	0.63	0.31	0.86	0.47	0.03	0.50	0.22	0.78	0.99	0.05
	LO	L	0.79	0.48	0.94	0.07	0.01	0.84	0.54	0.96	0.03	0.01	0.52	0.23	0.79	0.92	0.02
Sdq	GO	G	0.90	0.61	0.98	**0.01**	0.01	0.92	0.65	0.99	**0.00**	0.01	0.90	0.61	0.98	**0.01**	0.01
	GO	LO	0.59	0.28	0.85	0.62	0.05	0.61	0.29	0.86	0.53	0.03	0.61	0.29	0.86	0.53	0.05
	GO	L	0.70	0.39	0.89	0.23	0.01	0.64	0.33	0.87	0.41	0.02	0.70	0.39	0.89	0.23	0.01
	G	LO	0.33	0.11	0.65	0.32	0.01	0.24	0.07	0.58	0.14	0.01	0.33	0.11	0.65	0.32	0.01
	G	L	0.39	0.15	0.70	0.53	0.03	0.39	0.15	0.70	0.54	0.05	0.39	0.15	0.70	0.53	0.03
	LO	L	0.64	0.33	0.87	0.40	0.02	0.68	0.36	0.89	0.29	0.01	0.64	0.33	0.87	0.40	0.02
Sdr	GO	G	0.90	0.61	0.98	**0.01**	0.01	0.92	0.65	0.99	**0.00**	0.01	0.90	0.61	0.98	**0.01**	0.01
	GO	LO	0.61	0.29	0.86	0.53	0.03	0.63	0.31	0.87	0.46	0.05	0.61	0.29	0.86	0.53	0.03

	GO	L	0.70	0.39	0.89	0.23	0.01	0.66	0.35	0.88	0.34	0.01	0.70	0.39	0.89	0.23	0.01
	G	LO	0.33	0.11	0.65	0.32	0.01	0.27	0.08	0.60	0.19	0.01	0.33	0.11	0.65	0.32	0.01
	G	L	0.39	0.15	0.70	0.54	0.05	0.36	0.13	0.67	0.40	0.02	0.39	0.15	0.70	0.54	0.05
	LO	L	0.64	0.33	0.87	0.40	0.02	0.63	0.32	0.86	0.46	0.03	0.64	0.33	0.87	0.40	0.02
Sdv	GO	G	0.20	0.06	0.52	0.07	0.01	0.59	0.28	0.84	0.61	0.03	0.45	0.18	0.75	0.78	0.03
	GO	LO	0.02	0.00	0.21	**0.00**	0.01	0.33	0.11	0.66	0.33	0.01	0.37	0.13	0.69	0.46	0.01
	GO	L	0.23	0.07	0.56	0.11	0.02	0.45	0.19	0.74	0.76	0.05	0.57	0.28	0.82	0.67	0.01
	G	LO	0.27	0.08	0.59	0.17	0.03	0.24	0.07	0.56	0.13	0.01	0.51	0.21	0.81	0.96	0.05
	G	L	0.57	0.28	0.82	0.67	0.05	0.34	0.12	0.66	0.36	0.01	0.61	0.29	0.85	0.55	0.01
	LO	L	0.82	0.51	0.95	0.04	0.01	0.63	0.31	0.86	0.48	0.02	0.57	0.28	0.82	0.68	0.02
Sha	GO	G	0.35	0.12	0.67	0.39	0.01	0.41	0.16	0.72	0.61	0.02	0.41	0.16	0.72	0.60	0.02
	GO	LO	0.63	0.30	0.88	0.49	0.03	0.49	0.20	0.79	0.96	0.05	0.57	0.26	0.84	0.70	0.05
	GO	L	0.55	0.26	0.82	0.76	0.05	0.59	0.26	0.85	0.64	0.03	0.57	0.27	0.83	0.68	0.03
	G	LO	0.82	0.50	0.95	0.05	0.01	0.63	0.32	0.86	0.44	0.01	0.71	0.39	0.91	0.21	0.01
	G	L	0.70	0.38	0.89	0.23	0.01	0.71	0.40	0.90	0.19	0.01	0.64	0.33	0.87	0.40	0.01
	LO	L	0.36	0.13	0.67	0.40	0.02	0.64	0.33	0.87	0.40	0.01	0.39	0.15	0.70	0.54	0.01
Shv	GO	G	0.39	0.15	0.70	0.52	0.01	0.59	0.28	0.84	0.61	0.05	0.35	0.12	0.67	0.38	0.01
	GO	LO	0.55	0.26	0.81	0.77	0.03	0.78	0.44	0.94	0.11	0.01	0.55	0.24	0.82	0.79	0.05
	GO	L	0.59	0.28	0.84	0.62	0.02	0.70	0.38	0.90	0.24	0.01	0.64	0.33	0.87	0.40	0.02
	G	LO	0.65	0.33	0.88	0.39	0.01	0.73	0.40	0.92	0.18	0.01	0.73	0.38	0.92	0.21	0.01
	G	L	0.75	0.42	0.93	0.15	0.01	0.59	0.30	0.83	0.59	0.03	0.68	0.36	0.89	0.29	0.01
	LO	L	0.52	0.23	0.79	0.92	0.05	0.39	0.16	0.69	0.53	0.02	0.45	0.18	0.74	0.76	0.03
Sku	GO	G	0.63	0.31	0.87	0.47	0.03	0.73	0.39	0.92	0.19	0.01	0.73	0.41	0.92	0.17	0.01
	GO	LO	0.35	0.12	0.67	0.39	0.01	0.31	0.10	0.63	0.26	0.02	0.37	0.14	0.68	0.45	0.02
	GO	L	0.48	0.21	0.77	0.92	0.05	0.50	0.22	0.78	0.99	0.05	0.48	0.21	0.76	0.92	0.05
	G	LO	0.22	0.06	0.58	0.14	0.01	0.18	0.04	0.52	0.07	0.01	0.24	0.06	0.61	0.18	0.01
	G	L	0.34	0.12	0.65	0.35	0.01	0.29	0.09	0.61	0.21	0.01	0.18	0.05	0.50	0.05	0.01
	LO	L	0.64	0.33	0.87	0.41	0.02	0.64	0.33	0.87	0.41	0.03	0.63	0.30	0.87	0.50	0.03
Smc	GO	G	0.43	0.16	0.74	0.70	0.03	0.76	0.39	0.94	0.18	0.01	0.53	0.23	0.81	0.87	0.05
	GO	LO	0.63	0.32	0.86	0.44	0.02	0.61	0.29	0.86	0.55	0.01	0.59	0.28	0.84	0.60	0.01
	GO	L	0.73	0.42	0.91	0.15	0.01	0.71	0.38	0.91	0.24	0.01	0.71	0.39	0.91	0.22	0.01
	G	LO	0.71	0.39	0.91	0.22	0.01	0.49	0.20	0.78	0.96	0.05	0.59	0.28	0.84	0.61	0.02
	G	L	0.73	0.39	0.92	0.20	0.01	0.52	0.24	0.79	0.92	0.03	0.71	0.40	0.90	0.19	0.01
	LO	L	0.54	0.24	0.81	0.84	0.05	0.59	0.29	0.83	0.60	0.02	0.59	0.27	0.85	0.63	0.03
Smr	GO	G	0.40	0.15	0.71	0.56	0.01	0.48	0.19	0.79	0.92	0.03	0.35	0.11	0.69	0.43	0.01
	GO	LO	0.29	0.09	0.62	0.23	0.01	0.48	0.20	0.77	0.91	0.02	0.12	0.03	0.42	0.02	0.01
	GO	L	0.32	0.11	0.64	0.30	0.01	0.50	0.22	0.78	0.99	0.05	0.20	0.06	0.50	0.05	0.01
	G	LO	0.43	0.17	0.73	0.69	0.02	0.46	0.19	0.76	0.82	0.01	0.53	0.22	0.82	0.88	0.03
	G	L	0.52	0.24	0.79	0.92	0.05	0.46	0.20	0.75	0.84	0.01	0.54	0.24	0.81	0.85	0.02
	LO	L	0.54	0.25	0.80	0.84	0.03	0.55	0.26	0.81	0.76	0.01	0.52	0.24	0.79	0.92	0.05
Sp	GO	G	0.80	0.45	0.95	0.10	0.01	0.76	0.39	0.94	0.18	0.01	0.92	0.65	0.99	**0.00**	0.01
	GO	LO	0.53	0.22	0.82	0.88	0.03	0.59	0.27	0.85	0.62	0.01	0.57	0.24	0.85	0.73	0.03
	GO	L	0.75	0.41	0.93	0.16	0.01	0.75	0.42	0.92	0.14	0.01	0.79	0.44	0.94	0.11	0.01
	G	LO	0.37	0.13	0.70	0.48	0.02	0.49	0.20	0.78	0.96	0.05	0.39	0.14	0.71	0.55	0.02
	G	L	0.48	0.21	0.77	0.92	0.05	0.55	0.26	0.81	0.76	0.03	0.52	0.24	0.79	0.92	0.05
	LO	L	0.70	0.38	0.89	0.23	0.01	0.57	0.27	0.82	0.68	0.02	0.66	0.35	0.87	0.33	0.01
Spc	GO	G	0.86	0.56	0.97	0.02	0.01	0.84	0.53	0.96	0.03	0.01	0.88	0.59	0.97	0.01	0.01
	GO	LO	0.55	0.24	0.83	0.79	0.03	0.55	0.23	0.84	0.80	0.03	0.55	0.24	0.83	0.79	0.03
	GO	L	0.77	0.45	0.93	0.10	0.01	0.79	0.48	0.94	0.07	0.01	0.77	0.45	0.93	0.10	0.01
	G	LO	0.41	0.15	0.73	0.62	0.02	0.37	0.13	0.69	0.46	0.02	0.41	0.15	0.73	0.62	0.02

	G	L	0.52	0.24	0.79	0.92	0.05	0.50	0.22	0.78	0.99	0.05	0.52	0.24	0.79	0.92	0.05
	LO	L	0.64	0.33	0.87	0.40	0.01	0.70	0.38	0.89	0.23	0.01	0.63	0.31	0.86	0.47	0.01
Spd	GO	G	0.65	0.33	0.88	0.39	0.01	0.69	0.37	0.90	0.26	0.01	0.63	0.31	0.87	0.46	0.02
	GO	LO	0.39	0.14	0.72	0.55	0.03	0.43	0.16	0.74	0.70	0.02	0.35	0.11	0.69	0.42	0.01
	GO	L	0.43	0.17	0.73	0.69	0.05	0.46	0.19	0.76	0.85	0.03	0.38	0.14	0.69	0.48	0.03
	G	LO	0.18	0.05	0.50	0.05	0.01	0.16	0.04	0.49	0.04	0.01	0.18	0.05	0.50	0.05	0.01
	G	L	0.32	0.12	0.63	0.28	0.01	0.25	0.08	0.57	0.14	0.01	0.36	0.13	0.67	0.40	0.01
	LO	L	0.61	0.31	0.84	0.52	0.02	0.48	0.21	0.76	0.92	0.05	0.61	0.30	0.84	0.53	0.05
Sq	GO	G	0.43	0.17	0.74	0.70	0.05	0.61	0.30	0.85	0.52	0.02	0.80	0.47	0.95	0.08	0.01
	GO	LO	0.61	0.30	0.85	0.52	0.02	0.69	0.37	0.90	0.26	0.01	0.73	0.41	0.92	0.17	0.01
	GO	L	0.71	0.41	0.90	0.18	0.01	0.73	0.42	0.91	0.15	0.01	0.73	0.42	0.91	0.15	0.01
	G	LO	0.67	0.34	0.89	0.33	0.01	0.57	0.27	0.83	0.69	0.05	0.47	0.20	0.76	0.87	0.03
	G	L	0.79	0.46	0.94	0.09	0.01	0.63	0.32	0.85	0.46	0.01	0.52	0.23	0.79	0.92	0.05
	LO	L	0.59	0.28	0.84	0.61	0.03	0.57	0.28	0.82	0.67	0.03	0.61	0.30	0.85	0.53	0.02
Ssk	GO	G	0.67	0.34	0.89	0.33	0.03	0.82	0.45	0.96	0.09	0.01	0.88	0.58	0.97	0.02	0.01
	GO	LO	0.33	0.11	0.65	0.32	0.01	0.31	0.10	0.64	0.27	0.02	0.33	0.11	0.66	0.33	0.02
	GO	L	0.34	0.13	0.65	0.33	0.02	0.55	0.25	0.82	0.77	0.05	0.43	0.18	0.72	0.67	0.05
	G	LO	0.14	0.03	0.47	0.03	0.01	0.16	0.04	0.49	0.04	0.01	0.16	0.04	0.51	0.06	0.01
	G	L	0.16	0.04	0.48	0.04	0.01	0.20	0.06	0.50	0.05	0.01	0.18	0.05	0.49	0.05	0.01
	LO	L	0.43	0.18	0.72	0.67	0.05	0.64	0.33	0.87	0.41	0.03	0.64	0.33	0.87	0.40	0.03
Std	GO	G	0.63	0.32	0.86	0.45	0.02	0.43	0.16	0.74	0.70	0.05	0.57	0.27	0.83	0.69	0.05
	GO	LO	0.63	0.31	0.87	0.46	0.03	0.63	0.31	0.87	0.46	0.01	0.59	0.27	0.85	0.62	0.03
	GO	L	0.77	0.46	0.93	0.09	0.01	0.59	0.28	0.84	0.61	0.02	0.71	0.40	0.90	0.20	0.01
	G	LO	0.59	0.28	0.84	0.60	0.05	0.73	0.41	0.92	0.16	0.01	0.59	0.28	0.84	0.60	0.02
	G	L	0.71	0.40	0.90	0.19	0.01	0.57	0.28	0.82	0.67	0.03	0.70	0.39	0.89	0.23	0.01
	LO	L	0.68	0.36	0.89	0.29	0.01	0.38	0.14	0.68	0.46	0.01	0.64	0.34	0.86	0.39	0.01
Str	GO	G	0.67	0.35	0.89	0.32	0.01	0.78	0.43	0.94	0.13	0.01	0.57	0.27	0.83	0.69	0.01
	GO	LO	0.65	0.33	0.88	0.38	0.01	0.65	0.32	0.88	0.41	0.03	0.43	0.17	0.73	0.69	0.01
	GO	L	0.73	0.40	0.92	0.18	0.01	0.77	0.44	0.93	0.11	0.01	0.52	0.23	0.79	0.92	0.05
	G	LO	0.51	0.22	0.79	0.96	0.05	0.31	0.10	0.64	0.29	0.02	0.39	0.15	0.70	0.52	0.01
	G	L	0.63	0.31	0.86	0.47	0.02	0.50	0.22	0.78	0.99	0.05	0.46	0.20	0.75	0.84	0.02
	LO	L	0.59	0.29	0.83	0.60	0.03	0.71	0.40	0.90	0.20	0.01	0.54	0.24	0.81	0.85	0.03
Sv	GO	G	0.61	0.27	0.87	0.57	0.03	0.65	0.32	0.88	0.41	0.02	0.67	0.34	0.89	0.33	0.03
	GO	LO	0.71	0.39	0.91	0.21	0.01	0.73	0.41	0.92	0.17	0.01	0.82	0.48	0.96	0.07	0.01
	GO	L	0.77	0.45	0.93	0.10	0.01	0.82	0.52	0.95	0.04	0.01	0.82	0.51	0.95	0.04	0.01
	G	LO	0.59	0.27	0.85	0.63	0.05	0.57	0.25	0.84	0.71	0.05	0.76	0.42	0.93	0.14	0.02
	G	L	0.79	0.47	0.94	0.08	0.01	0.77	0.46	0.93	0.09	0.01	0.75	0.44	0.92	0.13	0.01
	LO	L	0.66	0.35	0.88	0.34	0.02	0.63	0.31	0.86	0.48	0.03	0.54	0.25	0.80	0.84	0.05
Sxp	GO	G	0.37	0.13	0.69	0.46	0.02	0.43	0.17	0.74	0.70	0.03	0.76	0.44	0.93	0.13	0.01
	GO	LO	0.57	0.27	0.83	0.68	0.05	0.61	0.30	0.85	0.52	0.02	0.73	0.40	0.92	0.18	0.01
	GO	L	0.73	0.42	0.91	0.16	0.01	0.68	0.36	0.89	0.29	0.01	0.95	0.69	0.99	**0.00**	0.01
	G	LO	0.73	0.41	0.92	0.16	0.01	0.73	0.41	0.92	0.17	0.01	0.51	0.22	0.79	0.96	0.05
	G	L	0.88	0.57	0.97	0.02	0.01	0.80	0.47	0.95	0.08	0.01	0.71	0.40	0.90	0.19	0.02
	LO	L	0.63	0.31	0.86	0.48	0.03	0.55	0.26	0.81	0.76	0.05	0.64	0.33	0.87	0.41	0.03
Sz	GO	G	0.86	0.54	0.97	0.03	0.01	0.82	0.50	0.95	0.05	0.01	0.84	0.53	0.96	0.03	0.01
	GO	LO	0.57	0.24	0.85	0.73	0.02	0.65	0.32	0.88	0.41	0.01	0.65	0.32	0.88	0.41	0.01
	GO	L	0.79	0.46	0.94	0.09	0.01	0.80	0.49	0.95	0.06	0.01	0.79	0.46	0.94	0.09	0.01
	G	LO	0.45	0.18	0.76	0.79	0.03	0.51	0.21	0.80	0.96	0.05	0.53	0.23	0.81	0.87	0.05
	G	L	0.54	0.25	0.80	0.84	0.05	0.63	0.32	0.86	0.47	0.02	0.59	0.29	0.83	0.60	0.03

	LO	L	0.64	0.33	0.87	0.40	0.01	0.59	0.29	0.83	0.60	0.03	0.59	0.29	0.83	0.60	0.02
Vm	GO	G	0.69	0.37	0.90	0.26	0.01	0.86	0.53	0.97	0.03	0.01	0.84	0.51	0.96	0.04	0.01
	GO	LO	0.43	0.17	0.73	0.68	0.03	0.39	0.14	0.71	0.53	0.03	0.67	0.35	0.89	0.32	0.02
	GO	L	0.54	0.25	0.80	0.84	0.05	0.59	0.29	0.83	0.60	0.05	0.70	0.39	0.89	0.23	0.01
	G	LO	0.18	0.04	0.52	0.07	0.01	0.14	0.03	0.50	0.05	0.01	0.29	0.09	0.61	0.21	0.01
	G	L	0.32	0.11	0.63	0.29	0.01	0.30	0.11	0.62	0.24	0.01	0.41	0.16	0.71	0.60	0.05
	LO	L	0.61	0.31	0.84	0.52	0.02	0.64	0.34	0.86	0.39	0.02	0.61	0.31	0.84	0.52	0.03
Vmc	GO	G	0.39	0.14	0.71	0.55	0.03	0.37	0.12	0.70	0.49	0.03	0.53	0.23	0.81	0.87	0.05
	GO	LO	0.63	0.32	0.86	0.44	0.02	0.67	0.35	0.89	0.32	0.02	0.61	0.30	0.85	0.52	0.01
	GO	L	0.75	0.43	0.92	0.13	0.01	0.73	0.42	0.91	0.16	0.01	0.73	0.40	0.92	0.18	0.01
	G	LO	0.78	0.45	0.94	0.10	0.01	0.80	0.47	0.95	0.08	0.01	0.59	0.28	0.84	0.61	0.02
	G	L	0.75	0.41	0.93	0.16	0.01	0.70	0.36	0.90	0.27	0.01	0.80	0.49	0.95	0.06	0.01
	LO	L	0.52	0.23	0.79	0.92	0.05	0.52	0.23	0.79	0.92	0.05	0.59	0.27	0.85	0.63	0.03
Vmp	GO	G	0.69	0.37	0.90	0.26	0.01	0.86	0.53	0.97	0.03	0.01	0.84	0.51	0.96	0.04	0.01
	GO	LO	0.43	0.17	0.73	0.68	0.03	0.39	0.14	0.71	0.53	0.03	0.67	0.35	0.89	0.32	0.02
	GO	L	0.54	0.25	0.80	0.84	0.05	0.59	0.29	0.83	0.60	0.05	0.70	0.39	0.89	0.23	0.01
	G	LO	0.18	0.04	0.52	0.07	0.01	0.14	0.03	0.50	0.05	0.01	0.29	0.09	0.61	0.21	0.01
	G	L	0.32	0.11	0.63	0.29	0.01	0.30	0.11	0.62	0.24	0.01	0.41	0.16	0.71	0.60	0.05
	LO	L	0.61	0.31	0.84	0.52	0.02	0.64	0.34	0.86	0.39	0.02	0.61	0.31	0.84	0.52	0.03
Vv	GO	G	0.43	0.16	0.74	0.70	0.03	0.49	0.21	0.78	0.96	0.05	0.57	0.27	0.83	0.69	0.03
	GO	LO	0.63	0.32	0.86	0.44	0.02	0.67	0.36	0.88	0.31	0.02	0.65	0.34	0.87	0.37	0.01
	GO	L	0.73	0.42	0.91	0.15	0.01	0.73	0.42	0.91	0.16	0.01	0.75	0.42	0.92	0.14	0.01
	G	LO	0.69	0.36	0.90	0.28	0.01	0.71	0.39	0.91	0.22	0.01	0.59	0.28	0.84	0.61	0.02
	G	L	0.71	0.37	0.91	0.24	0.01	0.70	0.36	0.90	0.28	0.01	0.71	0.40	0.90	0.19	0.01
	LO	L	0.55	0.26	0.81	0.76	0.05	0.54	0.24	0.81	0.84	0.03	0.55	0.25	0.82	0.77	0.05
Vvc	GO	G	0.47	0.19	0.77	0.87	0.05	0.49	0.21	0.78	0.96	0.05	0.51	0.22	0.79	0.96	0.05
	GO	LO	0.71	0.40	0.91	0.20	0.01	0.71	0.39	0.91	0.21	0.01	0.61	0.30	0.85	0.52	0.01
	GO	L	0.68	0.37	0.89	0.29	0.01	0.70	0.38	0.90	0.24	0.01	0.70	0.38	0.90	0.24	0.01
	G	LO	0.71	0.37	0.91	0.24	0.01	0.71	0.38	0.91	0.23	0.01	0.59	0.28	0.84	0.61	0.02
	G	L	0.68	0.35	0.89	0.32	0.02	0.68	0.35	0.89	0.32	0.02	0.68	0.37	0.88	0.27	0.01
	LO	L	0.54	0.25	0.80	0.84	0.03	0.52	0.23	0.79	0.92	0.03	0.57	0.26	0.83	0.70	0.03
Vvv	GO	G	0.37	0.14	0.68	0.45	0.03	0.41	0.16	0.72	0.61	0.03	0.86	0.53	0.97	0.03	0.01
	GO	LO	0.57	0.27	0.83	0.68	0.05	0.57	0.26	0.83	0.70	0.05	0.76	0.42	0.93	0.14	0.01
	GO	L	0.77	0.46	0.93	0.10	0.01	0.79	0.47	0.94	0.07	0.01	0.93	0.64	0.99	**0.01**	0.01
	G	LO	0.65	0.33	0.88	0.39	0.02	0.61	0.30	0.85	0.52	0.02	0.49	0.20	0.78	0.96	0.05
	G	L	0.89	0.61	0.98	**0.01**	0.01	0.84	0.51	0.96	0.04	0.01	0.71	0.40	0.90	0.19	0.02
	LO	L	0.66	0.34	0.88	0.35	0.01	0.64	0.31	0.88	0.45	0.01	0.68	0.36	0.89	0.29	0.03
